# CA-discharge: Geo-Located Discharge Time Series for Mountainous Rivers in Central Asia

**DOI:** 10.1038/s41597-023-02474-8

**Published:** 2023-09-04

**Authors:** Beatrice Marti, Andrey Yakovlev, Dirk Nikolaus Karger, Silvan Ragettli, Aidar Zhumabaev, Abdul Wakil Wakil, Tobias Siegfried

**Affiliations:** 1 hydrosolutions GmbH, Zurich, Switzerland; 2 Swiss Federal Research Institute WSL, Birmensdorf, Switzerland; 3 ETH Zurich, Zurich, Switzerland

**Keywords:** Hydrology, Environmental sciences

## Abstract

We present a collection of 295 gauge locations in mountainous Central Asia with norm discharge as well as time series of river discharge from 135 of these locations collected from hydrological yearbooks in Central Asia. Time series have monthly, 10-day and daily temporal resolution and are available for different duration. A collection of third-party data allows basin characterization for all gauges. The time series data is validated using standard quality checks. Norm discharge is validated against literature values and by using a water balance approach. The novelty of the data consists in the combination of discharge time series and gauge locations for mountainous rivers in Central Asia which is not available anywhere else. The geo-located discharge time series can be used for water balance modelling and training of forecast models for river runoff in mountainous Central Asia.

## Background & Summary

**Fig. 1:**
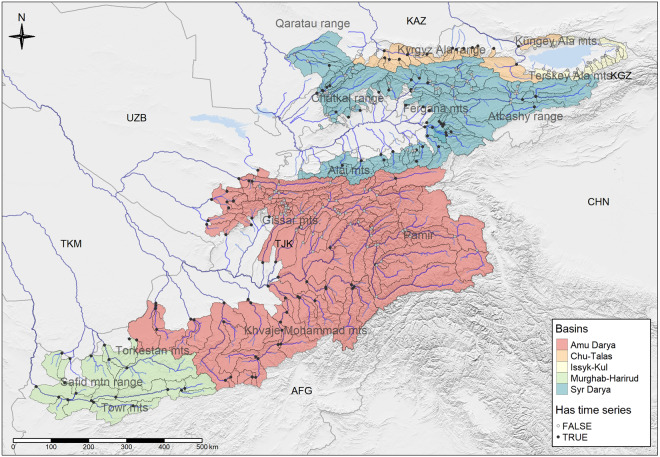
Overview over gauge locations and their respective catchment areas (slim grey lines) in Central Asia where discharge norms are available (light grey points) and where time series data is available (dark grey points). The background map is a hillshade layer derived from the DEM^14^. The clear blue polygons denote lakes^67^ and the dark blue lines indicate rivers^68^. Administrative boundaries (GADM v.4.1, gadm.org) are shown as thick grey lines. Mountain ranges (grey labels) have been digitized manually.

While highly vulnerable with regard to water availability and its impacts^1,2^, Central Asia remains for all practical purposes a hydrologically data-scarce region^3,4^. Knowledge about river discharge is the basis of understanding a hydrological system and of performing adequate water management. Forecast models for river runoff rely heavily on observations of past discharge for model setup and calibration^5^ as well as for example for the validation of gridded discharge products^6^. The presented data set provides the, to date, most comprehensive publicly available collection of *in-situ* measured discharge for Central Asia, including Afghanistan (see Fig. 1). It further provides a full basin characterisation as well as monthly time series of average temperature and snow cover and of monthly precipitation sums for each basin. The data is available as geopackage and can be downloaded from Zenodo^7^. Codes used to process the data are available from the same location.

## Methods

This section describes how the third-party data has been processed (in sequence) to obtain the resulting data layers (see Table 1). For reproducibility, the steps and R scripts to arrive at the data set presented here are made available in a Zenodo repository^7^. The repository further includes more detailed step-by-step instructions for anyone wishing to reproduce the data set (see also chapter Usage notes).

### Hydrological data

In former Soviet Central Asia, water levels in rivers were monitored typically twice a day, generally by a nearby resident observer. Few stations were equipped with water level tape recorders. During low-flow periods in winter, one measurement per day may have been taken (for example at gauge Mullala on the Pskem River (Chirchiq River basin). During high-flow periods, more than 2 measurements per day may have been taken. The observation frequency at each gauge is documented in the hydrological yearbooks but may change from one year to the next which has an impact on statistics derived from the observations. To this date, the data is collected and communicated to the regional hydrometeorological office on a daily basis where it is copied to ledgers and sent to the national hydrometeorological office. We will refer to the national hydrometeorological services as Hydromets in this article. From these ledgers, data is copied and processed in relation to the various information products the national Hydromets provide (e.g. hydrological yearbooks, long-term norm discharge, used for discharge forecasting). The organizational charts and tasks of the individual Hydromet services vary slightly from country to country. For the data collection in this paper, data were available from Kazakh^8^, Kyrgyz^9^, Tajik^10^, and Uzbek Hydromets^11^ but not from Turkmen Hydromet. Nowadays, this data collection and processing process is being digitized but the integration of digital data into well-established workflows is slow.

Discharge data can be accessed in daily resolution from the hydrological yearbooks archived in the Hydromets. The data is publicly available but needs to be digitized manually. The Hydromets offer daily river discharge data in digital format for sale. The present discharge time series dataset relies mostly on decadal (10-days) or monthly data gathered and processed by the Hydromets and published in the hydrological yearbooks. Time series data is available for a subset of gauges in the present data set. For each gauge in the data set, the norm discharge has been obtained from the hydrological yearbooks. Each gauge is attributed a reference called SOURCE (see Table 2) indicating the source of the data.

Gauge levels are collected in Afghanistan 3 times a day in a form or in 15 minutes intervals at newer automatic stations. Through monthly flow meter measurements, the rating curve is updated and discharge is calculated. During the flood season, flow meter measurements are collected every 10 days if possible. The data is copied in the regional office to a spreadsheet and subsequently sent to the Ministry of Energy and Water in Kabul. Historical monthly data is available until approximately 1980 (included in the presented data set). Newer data that has been collected in Afghanistan after 2008 is not available here.

### Cleaning of time series data

The raw data consisted of many datasets in different formats and types. Irrelevant texts and duplicates were removed. Then, all discharge data were converted to the same type; dates were synchronized and changed to the same date format. All data sets were formatted into a long (narrow) format and combined together. To make sure the data were not erroneous, visual and manual checks of all individual time series were done at all steps of cleaning. All data preparation was done in R (r-project.org/). Gauge locations for which time series data is available have the flag $has{\rm{\_}}ts$ set to TRUE in the attribute table. The temporal resolution of the time series was determined (daily, 10-day, or monthly) (gauges attribute: $res$). The start and end of the time series were extracted and stored in the gauge attributes $ts\_start$ and $ts\_end$. The period between $ts\_start$ and $ts\_end$ is henceforward called the observation period. The total number of observations in the observation period is given in the attribute $n\_complete$. This number indicates the number of time steps at a given temporal resolution in an observation period. Included in $n\_complete$ is the number of missing observations in the observation period (attribute $n\_miss$). $n\_propmiss$ gives the proportion of missing observation in the entire available time series for each station and $n\_largestgap$ shows the length of the largest observation gap in years.

### Gap filling of time series data

Individual data gaps of length 1 were filled using linear interpolation in R by applying the function na.seadec of the imputeTS package^12^ with option linear. This lead to the imputation of 1 monthly value in time series of monthly resolution, 1 10-day average value in time series of 10-day resolution and 1 daily value in time series of daily resolution. Longer gaps were left as are. Gap-filling resulted in a median change of the long-term norm discharge and mean monthly discharge of 0% and a mean change of 0.2% which was deemed acceptable.

### Gauge locations

As there are no geolocated consistent records of gauging stations available, all gauging stations were manually located in a Geographic Information System (GIS). In the former Soviet region, parts of gauging station names were often consisting of the village names where they are (were) located. For this reason, we developed a workflow to manually match station location names with village names found on the relevant Topographic maps (1:200’000) from the corresponding region and along the corresponding rivers. The topographic maps of the entire region were downloaded from https://maps.vlasenko.net and subsequently manually georeferenced in QGIS (qgis.org). Gauge locations were then inferred by visually inspecting high-resolution optical remote sensing imagery by locating obvious measurement locations, such as bridges or installations that would allow for cross-section measurements of water depth and velocity. The gauge locations are provided in the gauges layer.

### Catchment boundaries

The delineation of the catchment boundaries was done using WhiteboxTools v2.0.0^13^ which allows the concurrent delineation of watersheds from multiple gauge points. The workflow requires the gauge locations and a digital elevation model (DEM) as input. Here we use the SRTM DEM Global 1 arc second product^14^. The catchment delineation can be reproduced using the script $WatershedDelineation\_MultiplePourPoints\_V2.Rmd$ in the Zenodo repository^7^ (see also usage notes). Some catchment boundaries are not correctly delineated by the automated process and had to be edited manually in QGIS and then merged to the rest of the basins, namely the basins of gauges with ID 60029 (Kassansay River/Кассансай, gauge Uryukty/Урюкты). The R package sf^15^ was used to derive the area of each basin (basins attribute: $area\_km2$) which was compared to the literature^16–18^ (see technical validation). The outlines of the catchment boundaries are provided in the GIS layer basins.gpkg.

### Extraction of basin characteristics

Third-party data has been downloaded and extracted to each basin using the R package exactextractr (https://github.com/isciences/exactextractr) for fast, efficient extraction of raster data on polygons (basin boundaries in the present case). All third-party data is publicly available for free. For storage space reasons, we do not provide third-party data in this data repository but only the extracted results, i.e. average values for each basin. We do, however, provide the sources of each data type and detailed instructions on how to download and process the third-party data. The corresponding R script *extract_and_compile_catchment_data. Rmd* is available on Zenodo^7^ (see usage notes). A list of third-party data available for each basin is given in Table 3. The paragraphs below describe how the third-party data in the basins layer is derived.

### Statistics derived from DEM

The 30-arc-second global SRTM DEM^14^ was used as the basis to derive average characteristic parameters for each basin. The average basin slope (basin attribute: $slope$) and aspect (basin attribute: $aspect$) have been computed using the terrain function of the R package raster (http://CRAN.R-project.org/package=raster) with a method suitable for rough surfaces^19^. The Terrain Ruggedness Index (TRI, basin attribute: $tri$) is the basin average over the mean of the absolute differences between the value of a cell and the value of its 8 surrounding cells. The Topographic Position Index (TPI, basin attribute: $tpi$) is the basin average over the difference between the value of a cell and the mean value of its 8 surrounding cells. Roughness (basin attribute: $roughness$) is the basin average over the difference between the maximum and the minimum value of a cell and its 8 surrounding cells. $tri$, $tpi$ and $roughness$ are calculated using the raster package^20^. The attribute $flowdir$ contains the basin average of the direction of the largest difference between a cell and its neighbors. It corresponds to the average flow direction in the basin.

### Climate data

CHELSA v2.1 is a high-resolution (30 arc seconds or approx. 1 km grid resolution) climate data set. CHELSA v2.1 daily precipitation and temperature^21–23^ between 1981 and 2010 have been cut to the Central Asian domain. CHELSA temperature fields are produced using statistical downscaling from ERA5 temperatures and CHELSA precipitation fields incorporate orographic predictors and bias correction with station data from GPCC^21^. Daily average precipitation and temperature time series have been extracted for each basin and aggregated to monthly values. CHELSA v.2.1 climatologies^21,22^ have been downloaded and average climatologies have been extracted for each basin.

Monthly time series of average basin precipitation sums and mean basin temperature are calculated from daily values and included in the basin layer with attribute names *pr* or *tas* for precipitation or temperature respectively, followed by $\_mon\_$ and ending with the year and the month of the year as numbers, separated with an underline.

Bioclimatic indicators of the CHELSA v2.1 data set^21^ are extracted as averages per basin. The bioclimatic indicators are labelled with a prefix of $bio\_$ and a counter from 1 to 19 indicating the id of the bioclimatic variable as given by Karger and colleagues^21^. The indicators include: Annual mean temperature (bio1), mean diurnal range (bio2), isothermality (bio3), temperature seasonality (bio4), the maximum temperature of the warmest month (bio5), minimum temperature of the coldest month (bio6), temperature annual range (bio7), mean temperature of wettest quarter (bio8), mean temperature of driest quarter (bio9), mean temperature of warmest quarter (bio10), mean temperature of coldest quarter (bio11), annual precipitation (bio12), precipitation of wettest month (bio13), precipitation of driest month (bio14), precipitation seasonality (bio15), precipitation of wettest quarter (bio16), precipitation of driest quarter (bio17), precipitation of warmest quarter (bio18), precipitation of coldest quarter (bio19). Thereby a quarter corresponds to one-fourth of a year, i.e. 3 months. Further, the basin layer includes growth season statistics, for example, the first and last days of the growing season based on the daily temperature and precipitation fields in CHELSA that can be used for the estimation of tree line position (TREELIM)^24^. Further, the bioclimatic indicators include an estimate of net primary production made with Lieths Miami model^25^. The parameters are described in detail in the CHELSA v2.1 technical manual^26^. A brief description of the variables is given in the basin description table included in the data set presented here (layer $basin\_attribute\_description$ in the geopackage).

The data set further includes classifications of climatologies according to Köppen-Geiger^27^ (basin attributes: $kg0$ and $kg1$), modified Köppen-Geiger^28^ ($kg2$), climatologies according to Wissmann^29^ ($kg3$) and Thornthwaite^30^ ($kg4$) and Troll and Paffen^31^ ($kg5$).

For comparison with the CHELSA climatologies, average basin WorldClim climatologies between 1970 and 2000^32^ are provided in the basin layer. The attributes include average annual norm precipitation as well as average annual cold and warm season precipitation (attributes: $pr\_worldclim\_ann$, $pr\_worldclim\_cs$ and $pr\_worldclim\_ws$ respectively). Further global data sets have been included for comparison: The CHELSA W5E5 precipitation product^33^ (basin attribute: $pr\_ann\_w5e5$); the PBCorr precipitation product^23^ which includes bias corrections for the following data sets: CHPclim V1^34^ (basin attribute: $pr\_ann\_chp$) and WorldClim v21^32^ (basin attribute: $pr\_ann\_worldclim$); and the average annual evaporation and aridity index between 1970 and 2000 (CGIAR)^35,36^ (basin attributes: $pet\_ann\_cgiar$, $pet\_cs\_cgiar$, $pet\_ws\_cgiar$ and $ai\_ann\_cgiar$).

### Snow cover

Based on the daily pixel values of CHELSA v2.1 temperature, daily snow cover fractions are estimated^37^, aggregated to annual data and averaged over the period between 1981 and 2010. The average annual snow cover fraction in per basin is given in the basin attribute $fs\_ann\_chelsa$.

Monthly mean snow covered fraction of basin area between January 2000 and December 2021 was extracted from the daily snow cover products (MOD10A1 and MYD10A1) of Moderate-Resolution Imaging Spectroradiometer (MODIS) satellite imagery^38^ (basin attribute: $scf\_ < YEAR > \_ < MONTH >$). We use the MODIS snow cover mapping based on the Normalized Difference Snow Index (NDSI), which reveals the magnitude of the difference between reflectance in visible bands and in the shortwave infrared, respectively. A high difference is typical for snow. We use the $NDSI\_Snow\_Cover$ band, which represents the Snow Cover Fraction (SCF) at the subpixel level within 500 m grid cells^39^. For the combination of the two MODIS products and for cloud-gap-filling we use the method as detailed in Tang *et al*.^40^. Maps and time-series of SCF from every study basin can be viewed and downloaded through an Earth Engine application (https://hydrosolutions.users.earthengine.app/view/snowcovermapper-ca).

### Land cover

Land cover data from the Copernicus 100 m 2019 land cover data^41^ set was downloaded and extracted for each basin. The basin attributes are names using the prefix $lc\_$ with the id number of the Copernicus land cover class. For example, $lc\_20$ for the basin area classified as shrubs in square kilometers. The class ids of the Copernicus land cover classification are taken from Buchhorn and colleagues^42^ and reproduced in the $basin\_attribute\_description$ layer. The complete list of land cover classes occurring in the presented data set include: 20 (Shrubs), 30 (herbaceous vegetation), 40 (cultivated and managed vegetation/agriculture), 50 (urban/built up), 60 (bare/sparse vegetation), 70 (snow and ice), 80 (open water), 90 (herbaceous wetland), 100 (moss and lichen), 111 (closed forest, evergreen needle leaf), 112 (closed forest, evergreen broad leaf), 113 (classified as closed forest, deciduous needle leaf), 114 (classified as closed forest, deciduous broad leaf), 115 (closed forest, mixed), 116 (closed forest, unknown), 121 (open forest, evergreen needle leaf), 122 (open forest, evergreen needle leaf), 123 (open forest, deciduous needle leaf), 124 (open forest, deciduous broad leaf), 125 (open forest, mixed), and 126 (open forest, unknown).

### Glacier storage

Glacier areas are extracted for each basin from the Randolph Glacier Inventory Version 6.0^43^ (basin attribute: $gl\_A\_km2$). The glacier volume ($gl\_V\_km3$) is estimated on a per-glacier basis using the empirical area volume scaling function by Erasov^44^ and aggregated for each basin. The fraction of glaciated area per basin (basin attribute: $gl\_fr$) is computed as $gl\_A\_km2/area\_km2$. Average glacier thinning rates as water equivalents per year and average annual glacier mass loss between 2000 and 2010 from Hugonnet and colleagues^45^ were extracted and aggregated by basin (basin attributes: $gl\_dmdtda\_mma$ and $gl\_dmdt\_km3a$).

### Mapping of gauge network

For each gauge within a river system, the code of the downstream gauge was manually added as attribute to the spatial basins layer (attribute name: $dnstr\_gauge$) in QGIS 3. Further, the sub-basins were manually assigned an attribute $basin\_order$ indicating if the catchment is a headwater basin ($basin\_order$ = 0) or a downstream basin ($basin\_order$ > 0). The higher the $basin\_order$, the more upstream basins drain through a downstream gauge. The mapping of the gauge network was validated in R using network functionalities from the GGally package (https://github.com/ggobi/ggally).

## Data Records

The GIS layers and discharge time series presented here are available as a geopackage through Zenodo^7^. Table 1 gives an overview of the available layers in the geopackage. Each layer is described in more detail in the following sub-sections. The presented data set includes a gauges layer with point features for each gauge location ($gauges$), a basins layer with the outlines of the catchment areas for each gauge location ($basins$), an attribute table with average basin characteristics ($basin\_attributes$), an attribute table description with detailed explanations for each basin attribute ($basin\_attribute\_description$), and a table with discharge time series data ($discharge\_time\_series$). The $gauges$ layer shows 297 features (or gauges) while the $BASIN$ and $basinattributes$ layers only show 295 features (or basins). This is due to the fact that for one gauge (station 17050 on the Gunt River) decadal as well as monthly time series are available and for another gauge (station 16070 on the Small Naryn River) daily and decadal data are available which are only partially overlapping. These two time series are marked in the gauges layer as well as in the discharge time series layer with codes 17050d and 17050m for the higher resolution and and lower resolution time series respectively. These codes are present also in the layer $discharge\_time\_series$. The $basins$ and $basins\_attributes$ use the station codes 17050 and 16070 respectively.

**Table 1:** Layer information for the geopackage CA-discharge.gpkg.

Layer name	Geometry type	Features	Fields	CRS
gauges	Point	297	20	WGS 84
basins	Polygon	295	8	WGS 84
basin_attributes	—	295	1107	—
quality_flags	—	297	11	—
basin_attribute_description	—	135	5	—
discharge_time_series	—	249826	4	—

The features and fields columns indicate the dimensions of the layers. CRS stands for Coordinate Reference System. No CRS is available for data layers without geometry. Data layers are linked to geometry layers through unique identifier attributes called CODE.

**Fig. 2:**
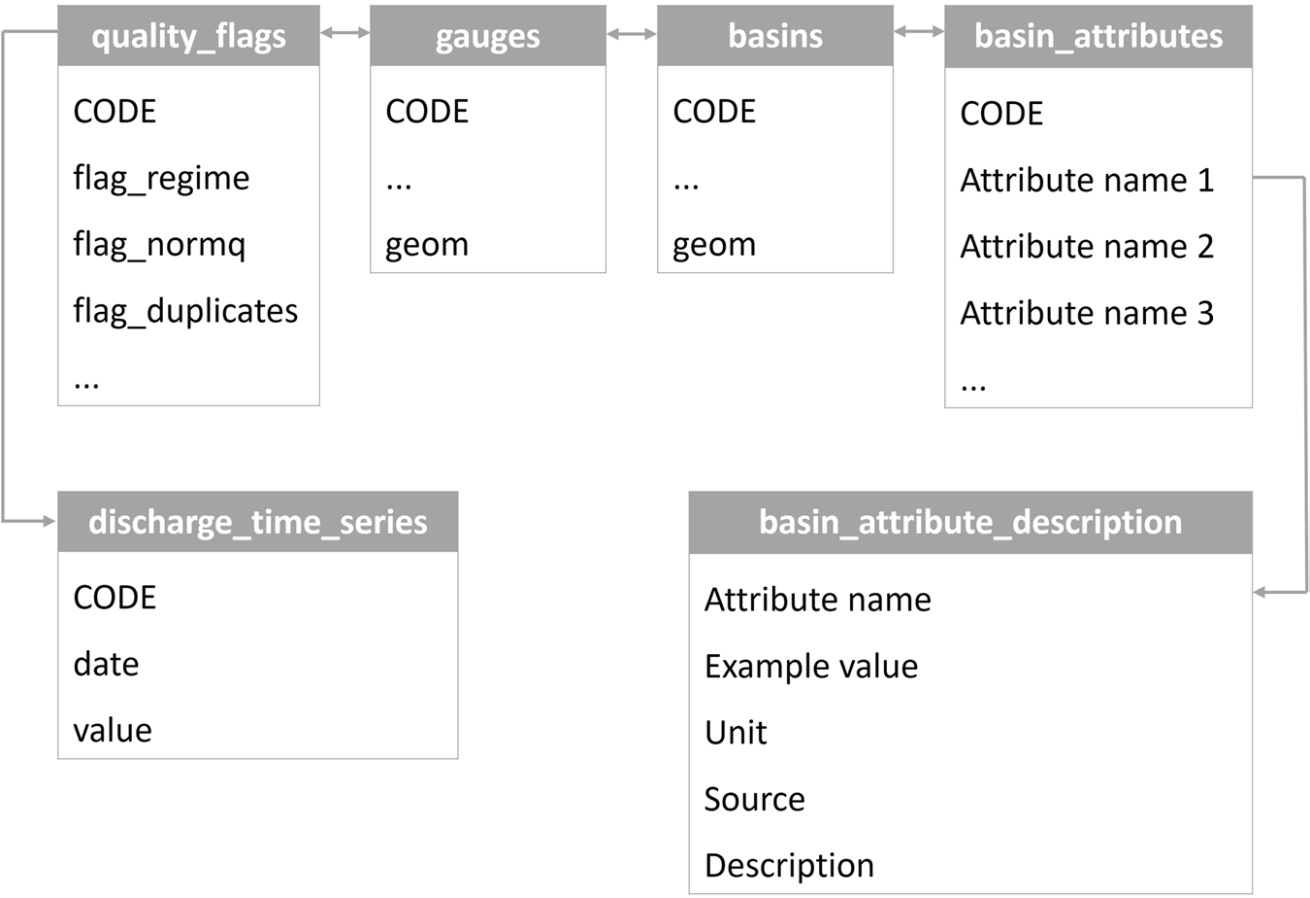
Table schema illustrating the linkages between the layers in the geopackage. The attribute *CODE* links gauges, basins, basin attributes as well as time series. Each gauge in the gauges layer is linked to at most one discharge time series. For each attribute in the $basin\_attributes$ layer, a detailed description is available in the $basin\_attribute\_description$ layer.

All layers are linked through a unique gauge ID (*CODE*). Each attribute in the $basin\_attributes$ layer is described in detail in the $basin\_attribute\_description$ layer. Figure 2 an overview over how the different tables can be cross-referenced through the $CODE$ field and through the attribute name as a database diagram.

### The gauges layer

Table 2 gives an overview of the gauge attributes included in the data set. All gauges are uniquely identified throughout the data set by their station codes (attribute $CODE$) which either consists of a 5-digit code or a combination of digits and characters as detailed hereafter. The 5-digit code starting with 15 refers to gauges in the basins of the Chu River and Talas River as well as of lake Issyk Kul. The number starts with 16 for gauges in the Syr Darya basin, and with 17 for gauges in the Amu Darya basin^8–11^. For some gauges, it was not possible to identify the official 5-digit code of a station. This is the case for example for gauges which stopped measuring after independence, more than 30 years ago. For these gauges an arbitrary 5-digit code was assigned, starting with the basin identifier (5 for Chu, Talas and Issyk Kul, 6 for Syr Darya and 7 for Amu Darya) and ending with a digit identifying the gauge (e.g. 60003). Afghan stations consist of a first 1–2 digit number identifying the river, followed by a combination of 4 and 2 digits and characters, separated by a hyphen^46^. Gauge locations are available as geometry attributes in a WGS84 projection (EPSG:4326) ($geom$) as well as attributes in EPSG:4326 and EPSG:32642 (UTM 42 N) projections ($EASTING$, $NORTHING$, $LON$, $LAT$). English names for gauges are available as transliterations of Russian gauge names (where available)^8–11,16–18^. Russian gauge names are written in Cyrillic letters which require UTF-8 compatibility for display. The name of the river at which the gauge location is located is given in the attribute $name$.The attribute $COUNTRY$ contains the country within which boundaries the gauge location falls. The $BASIN$ attribute shows in which regional river basin the river is draining into. The long-term average or norm discharge is given in attribute $q\_m3s$. The attribute $Source$ indicates the source of the norm discharge value. The data sources include the following: USGS Data Report 529^46^, Kazakhhydromet^8^, Kyrgyzhydromet^9^, Tajikhydromet^10^, Uzbekhydromet^11^, Surface Water Resources, Vol. 14, Issue 1^16^, Surface Water Resources, Vol. 14, Issue 3^17^, and Yuri Ivanov^18^. The norm discharge is not calculated from time series data but taken from the aforementioned sources. The attribute $has\_ts$ is a TRUE or FALSE flag indicating if time series data is available for a gauge location. If $has\_ts$ is TRUE, the remaining attributes contain information about the temporal resolution of the time series data ($res$), the first and the last date of the time series ($ts\_start$ and $ts\_end$, also called the observation period), the total number of time steps at a given resolution in the observation period ($n\_complete$), the number of missing observations in the observation period ($n\_miss$), the proportion of missing values in the observation period ($n\_propmiss$) and the size of the largest data gap in a count of observations (*n_largestgap*). If $has\_ts$ is FALSE, the remaining attributes are empty.

**Table 2:** Attributes of the gauges layer.

Attribute name	Example value	Source	Description
CODE	16510, 10-0.000-3 M	Water resources compendia^16,17^; Ivanov, 2010^18^; National Hydromet organisations^8–11^; Olson, 2010^46^	A 5-digit number or a 10-11 digit code.
EASTING	731’737.97	Manual	Easting of the gauge location in UTM42N (EPSG:32642).
NORTHING	4’444’266.36	Manual	Northing of the gauge location in UTM42N (EPSG:32642).
LON	71.72	Manual	Longitude of the gauge location in degrees (EPSG:4326).
LAT	40.12	Manual	Latitude of the gauge location in degrees (EPSG:4326).
NAME_ENG	Koksu mouth	Transliteration	English name of the gauge, if available.
NAME_RU	Коксу устье	Water resources compendiums^16,17^; Ivanov, 2010^18^; National Hydromet organisations^8–11^	Russian name of the gauge, if available.
NAME	Koksu	Water resources compendia^16,17^; Ivanov, 2010^18^; National Hydromet organisations^8–11^	Name of the river.
COUNTRY	UZB	GADM	Contry the gauge is located in.
BASIN	SYR_DARYA	HydroAtlas^66^	Name of the river basin the gauged river is draining to.
q_m3s	2.4	Water resources compendiums^16,17^; Ivanov, 2010^18^; National Hydromet organisations^8–11^	Annual norm discharge in m^3^/s as reported by the monitoring organisation.
SOURCE	TAJ HM	Water resources compendiums^16,17^; Ivanov, 2010^18^; National Hydromet organisations^8–11^	Name of the data source.
res	month	Derived	Temporal resolution of the time series data.
has_ts	TRUE	Derived	Flag indicating if time series data is available for this station.
ts_start	1964-01-15	Derived	First date with observations.
ts_end	2010-12-25	Derived	Last date with observations.
n_complete	180	Derived	Total number of observations.
n_miss	11	Derived	Number of missing observations between ts_start and ts_end.
n_propmiss	0.06	Derived	n_miss/n_complete
n_largestgap	2	Derived	Largest data gap in a count of missing observations.
geom	—	Derived	Point geometry information.

### The basins layer

Each basin in the $basin$ layer is linked to the gauges in the $gauges$ layer through the attribute $CODE$. The $basins$ layer only includes a few basin attributes (see Table 3) to keep it at a reasonable size for visualizations, namely $CODE$, $BASIN$, $SOURCE$, $REGION$, $area\_km2$, $q\_m3s$, $q\_m3a$, and $q\_mm$. a more detailed description of these basin attributes is given in the layer $basin\_attribute\_description$ which is discussed below. The attributes $CODE$, $BASIN$, and $SOURCE$ are the same as in the $gauges$ layer. Further, the attribute $q\_m3s$ in the $basins$ and $basins\_attributes$ layer corresponds to the attribute $q\_m3s$ in the $gauges$ layer.

**Table 3:** Attributes of the basins layer.

Name	Description
CODE	A 5-digit number or a 10–11 digit code^8–11,16–18,46^
BASIN	Name of the river sub-basin the gauged river is draining to^66^.
SOURCE	Name of the data source^8–11,16–18,46^
REGION	Name of regional river basin the river is draining to^66^.
area_km2	The basin area in square kilometres^14,15^
q_m3s	Norm discharge in cubic metres per second^8–11,16–18,46^
q_m3a	Norm discharge in cubic metres per year^8–11,16–18,46^
q_mm	Specific river discharge (q_m3a/area_km2) of the basin in millimetres per year.

This table only gives an overview, please refer to the basin_attribute_description layer in the geopackage for more details.

### The basin attributes layer

The $basin\_attributes$ layer is linked to both $gauges$ and $basins$ layers through the $CODE$ attribute. Table 4 gives an overview of the attributes included in the basins attribute layer. For space reasons, this table remains descriptive. A detailed description, including data units and examples, is given in the layer $basin\_attributes\_description$ of the geopackage.

**Table 4:** Full list of attributes available in the basin_attributes data table layer.

Name	Description
CODE	A 5-digit number or a 10–11 digit code^8–11,16–18,46^
BASIN	Name of the river sub-basin the gauged river is draining to^66^.
EASTING	Easting of the gauge location in UTM42N (EPSG:32642).
NORTHING	Northing of the gauge location in UTM42N (EPSG:32642).
LON	Longitude of the gauge location in degrees (EPSG:4326).
LAT	Latitude of the gauge location in degrees (EPSG:4326).
SOURCE	Name of the data source^8–11,16–18,46^
REGION	Name of regional river basin the river is draining to^66^.
area_km2	The basin area in square kilometres^14,15^
q_m3s	Norm discharge in cubic metres per second^8–11,16–18,46^
q_m3a	Norm discharge in cubic metres per year^8–11,16–18,46^
q_mm	Specific river discharge (q_m3a/area_km2) of the basin in millimetres per year.
h_mean	Mean elevation of the basin in metres above mean sea level^14^.
h_min	Minimum elevation of the basin in metres above mean sea level^14^.
h_max	Maximum elevation of the basin in metres above mean sea level^14^.
slope	Mean slope in the basin^14,19^
aspect	Mean aspect in the basin^14,19^
tpi	Mean Topographic Position Index in the basin^14,20^
tri	Mean Terrain Ruggedness Index in the basin^14,20^
roughness	Mean topographical roughness^14,20^
flowdir	Mean flow direction in a basin^14^
lc_X	Copernicus land cover in km2 with X ranging from 20 to 126 according to the Copernicus land cover classes^41,42^
gl_A_km2	Glaciated area from the Randolph Glacier Inventory v6.0^43^
gl_V_km2	Glacier volume estimated using Erasov, 1968^44^
gl_fr	Fraction of glaciated area in total basin area
gl_dmdtda_mma	Basin average of per-glacier thinning rates in water equivalent by Hugonnet and colleagues^45^
gl_dmdt_km3a	Basin average of per-glacier mass loss rates in water equivalent by Hugonnet and colleagues^45^
fs_ann_chelsa	Snow cover fraction estimated from daily CHELSA v2.1^21,22^
ai_ann_bio_chelsa	Aridity index calculated from daily CHELSA v2.1^21,22^
bio1 till wi	Bioclimatic parameters included in CHELSA v2.1^26^.
pr_mon_X_Y	Sum monthly precipitation in mm/month for year X and month Y
tas_mon_X_Y	Average monthly temperature in deg C for year X and month Y
pr_ann_w5e5	Average annual precipitation from CHELSA W5E5^33^
pr_ann_chpclim	Average annual bias-corrected CHPclim precipitation from^23,34^
pr_ann_worldclim	Annual average bias-corrected Worldclim precipitation from^23,32^
X_ann_cgiar	Average annual X [pet: evaporation, ai: aridity index]^35,36^
scf_X_Y	Snow covered fraction *TODO citations from Silvan*
dnstr_gauge	Gauge code of downstream lying gauge along the river network.
basin_order	Number between 0 and 9 indicating where along a gauge network the gauge lies. 0 for headwater basins and higher numbers for downstream gauges.

This table only gives a short description of the basin attributes. Please refer to the basin_attribute_description layer in the geopackage for a complete description in table form.

The first basin attributes from $CODE$ to $SOURCE$ are identical to the gauge attributes. The attribute $REGION$ gives the name of the drainage basin of regional importance. The long-term average runoff in m^3^/s produced from the basin ($q\_m3s$) corresponds to the gauge attribute $QNORM\_M3S$ from which attributes $q\_m3a$ and $q\_mm$ are derived. Attributes derived from the DEM range from the basin area ($area\_km2$), over the basin averages of elevation ($h\_mean$), slope ($slope$), aspect ($aspect$), Topographic Position Index ($tpi$), Terrain Ruggedness Index ($tri$), topographical roughness ($roughness$) to the average flow direction in the basin ($flowdir$). Further, the basin minimum and maximum elevations are given in the attributes $h\_min$ and $h\_max$ respectively. Elevations are given in meters above mean sea level (masl). The areas of different land cover classes^41^ are given in attributes with the prefix *lc_*. A detailed description of each land cover class is given in the layer $basin\_attribute\_description$. The glacier related parameters are glacier area ($gl\_A\_km2$), glacier volume ($gl\_V\_km3$), fraction of glaciated area ($gl\_fr$), glacier thinning rates ($gl\_dmdtda\_mma$ and $gl\_dmdt\_km3a$)^45^. The average snow cover fraction of the basin is given in the attribute $fs\_ann\_chelsa$. Next the entire range of bioclimatic indicators available through CHELSA v2.1 is given as basin averages (indicated as $bio1$ til $wi$ in Table 4). These include the aridity index ($ai\_ann\_bio\_chelsa$), the annual mean temperature ($bio1$), mean diurnal range ($bio2$), isothermality ($bio3$), temperature seasonality (*bioi*4), the maximum temperature of the warmest month ($bio5$), minimum temperature of the coldest month ($bio6$), temperature annual range ($bio7$), mean temperature of wettest quarter ($bio8$), mean temperature of driest quarter ($bio9$), mean temperature of warmest quarter ($bio10$), mean temperature of coldest quarter ($bio11$), annual precipitation ($bio12$), precipitation of wettest month ($bio13$), precipitation of driest month ($bio14$), precipitation seasonality ($bio15$), precipitation of wettest quarter ($bio16$), precipitation of driest quarter ($bio17$), precipitation of warmest quarter ($bio18$), precipitation of coldest quarter ($bio19$). Thereby a quarter corresponds to one-fourth of a year, i.e. 3 months. Further attributes include the frost change frequency ($fcf$), the first day of the growing season ($fgd$), the growing degree days heat sum above 0, 5 and 10 degrees Celsius ($gdd0$, $gdd5$, $gdd10$), the first and the last growing degree day above 0, 5, or 10 degrees Celsius ($gdgfgd0$, $gdgfgd5$, $gdgfgd10$, $gdglfgd0$, $gdglfgd5$, $gdglfgd10$), the growing season length ($gsp$), the mean temperature of the growing season ($gst$), the accumulated precipitation in the growing season($gsp$), the maximum, mean, minimum, and range of relative humidity ($hur{s}_{m}ax$, $hur{s}_{m}ean$, $hur{s}_{m}in$, $hur{s}_{r}ange$), the climate classifications ($kg0$, $kg1$, $kg2$, $kg3$, $kg4$, $kg5$), the last growth day of the season ($lgd$), the number of days at which the daily average temperatures is above 0, 5 or 10 degrees Celsius ($ngd0$, $ngd5$, $ngd10$), the net primary production ($npp$), the maximum, mean, minimum and range of monthly potential evaporation, wind speed, total cloud cover, and vapor pressure deficit ($pet\_penman\_max$, $pet\_penman\_mean$, $pet\_penman\_min$, $pet\_penman\_range$, $sfcWind\_max$, $sfcWind\_mean$, $sfcWind\_min$, $sfcWind\_range$, $tcc\_max$, $tcc\_mean$, $tcc\_min$, $tcc\_range$, $vpd\_max$, $vpd\_mean$, $vpd\_min$, $vpd\_range$), the number of days with snow cover ($scd$), the average snow water equivalent ($swe$), and the wetness index ($wi$). The monthly time series of basin average precipitation and temperature are given in the attributes $pr\_mon\_ < YEAR > \_ < MONTH >$ and $tas\_mon\_ < YEAR > \_ < MONTH >$ from January 1981 to December 2012. The monthly snow cover fraction between January 2000 and December 2021 is given in attribute $scf\_ < YEAR > \_ < MONTH >$. Last but not least climatologies from other data products are available for comparison, namely the annual precipitation from CHELSA W5E5 V1 ($pr\_ann\_w5e5$), from CHPclim V1 ($pr\_ann\_chpclim$), and from WorldClim V21 ($pr\_ann\_worldclim$) and mean annual, cold and warm season potential evaporation from CGIAR ($pet\_ann\_cgiar$, $pet\_cs\_cgiar$, $pet\_ws\_cgiar$) as well as the CGIAR aridity index ($ai\_ann\_cgiar$).

### The quality flags layer

Validation of discharge time series, norm discharge as well as basin geometry is done. For each quality check that is done, a flag is written describing fail or pass of the test. The reader is referred to the technical validation section for a detailed description of each quality flag.

### The basin attribute description layer

The detailed description of the basin attributes is given in the $basin\_attribute\_description$ layer. For each attribute ($Attribute$ $name$), an example value is given ($Example$ $value$), the unit of the value ($Unit$), the data source ($Source$), and one to two sentences of description for each attribute ($Description$).

### The discharge time series layer

**Fig. 3:**
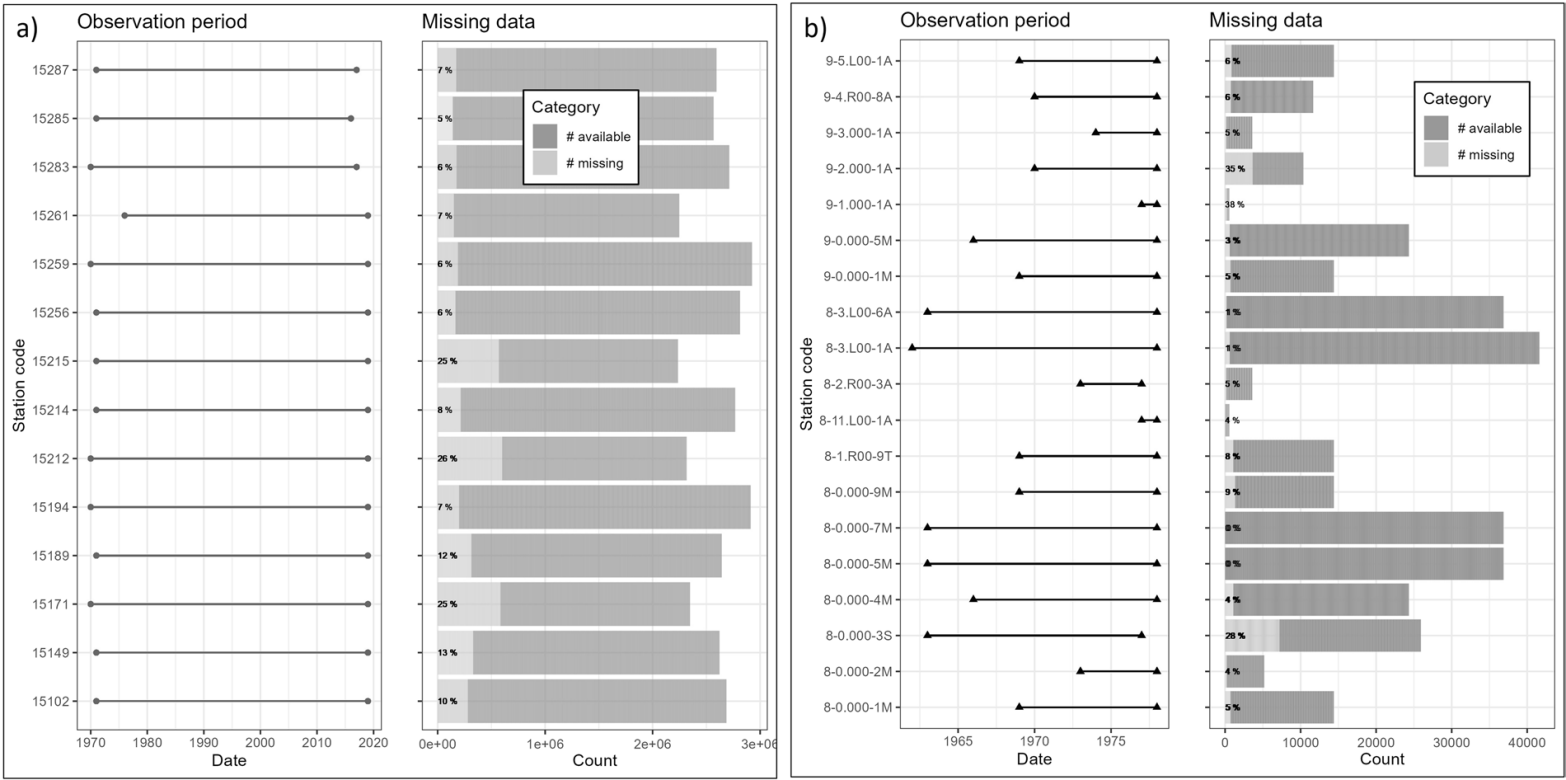
Time series data availability and gaps in the sub-basins of (**a**) Chu, Talas and Lake Issyk Kul and (**b**) Murghab and Harrirud. The Observation period is indicated by the available time series’ first and last dates. Daily data is indicated by light grey squares, monthly data is indicated by dark grey triangles, and decadal (10-day) data is indicated by medium grey circles in the observation period plot. The percentage of missing data has been calculated based on the observation period of each gauge and added as a label to the missing data plot.

**Fig. 4:**
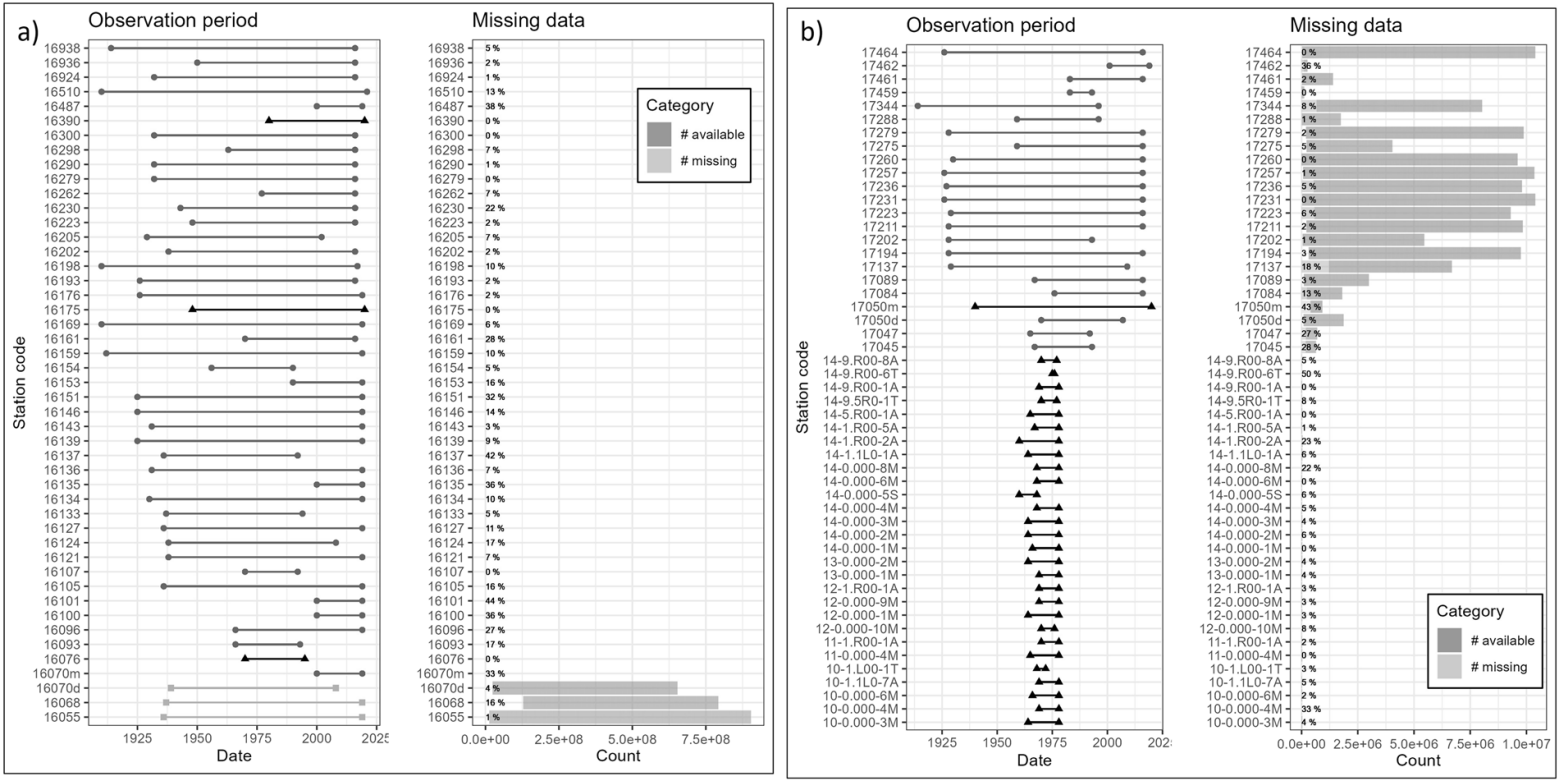
Time series data availability and gaps in the sub-basins of the (**a**) Syr Darya (**b**) Amu Darya rivers. The Observation period is indicated by the available time series’ first and last dates. Daily data is indicated by light grey squares, monthly data is indicated by dark grey triangles, and decadal (10-day) data is indicated by medium grey circles in the observation period plot. The percentage of missing data has been calculated based on the observation period of each gauge and added as a label to the missing data plot.

The $discharge\_time\_series$ layer includes dates (*date*) and discharge values in cubic meters per second (*value*) for each gauge location listed in the layer (*CODE*). Figures 3, 4 give an overview of the start and end of each available discharge time series as well as the number of data points and data gaps in each time series. The gauges are grouped per river basin in these Figures: Fig. 3 shows gauges in the basins of the rivers Chu, Talas, Harirud, and Murghab as well as of lake Issyk Kul. Figure 4 shows gauges in the basins of the Syr Darya and Amu Darya.

**Fig. 5:**
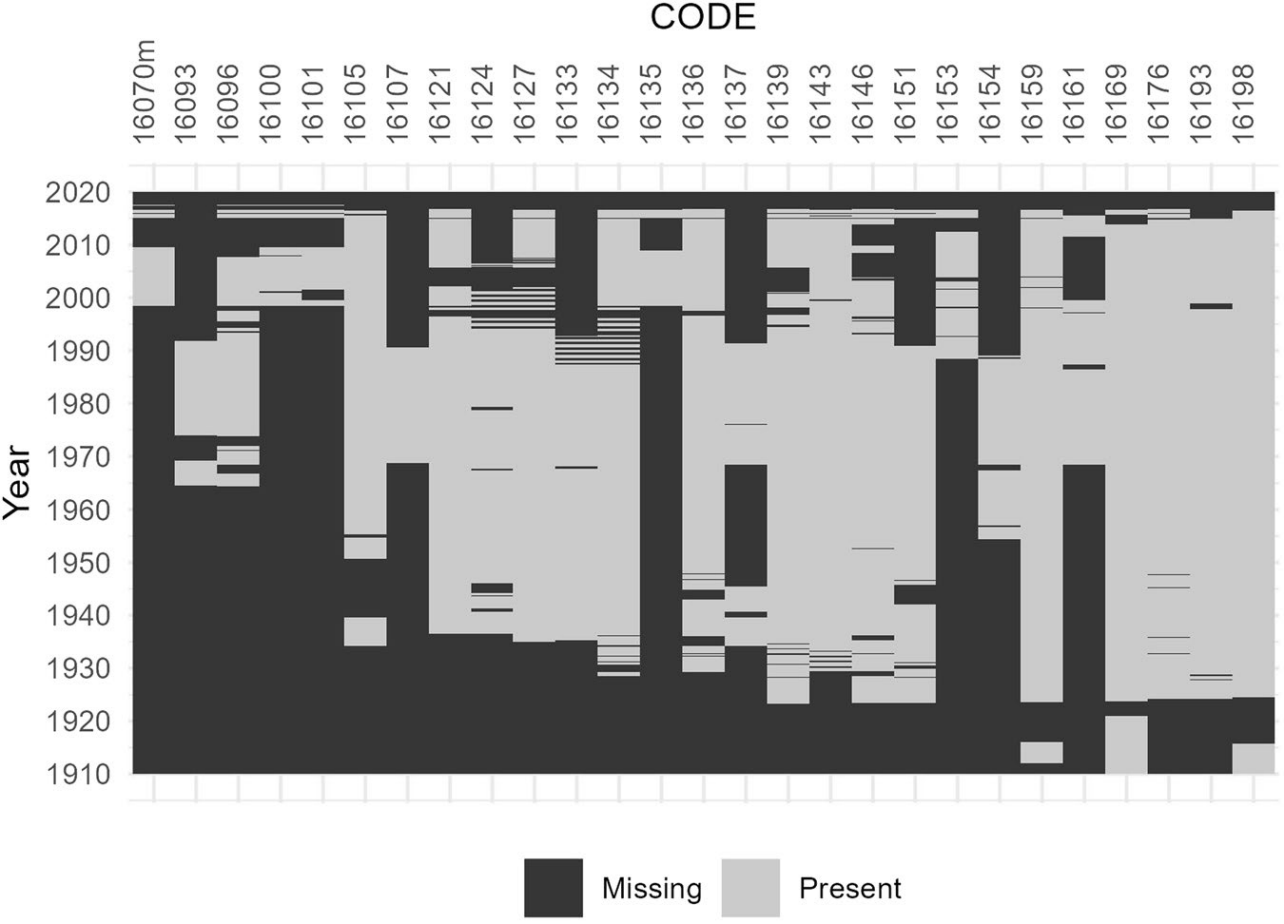
Example of data availability at 26 gauges from the Syr Darya basin with decadal observations. A change in the monitoring frequency or even the abandonment of gauges is visible in the 1990ies in many stations.

The time series from gauges in Afghanistan are only available in monthly time steps and end before 1979, i.e. before the Soviet-Afghan war. Data gaps are present in several gauge locations in the early 90es when, after the demise of the USSR, hydrological monitoring was interrupted (see for example Fig. 5). Most of the time series presented here go up to the year 2012. More recent data are available in the hydrological yearbooks.

The discharge time series overlapping with the CHELSA climatology data set between 1981 and 2010 include 83 gauges. However, only 5 discharge time series (CODE 15261 (Talas River/Талас, gauge Klyuchevka/Ключевка), 15285 (Ur-Maral River/Ур-Марал, gauge October/Октябрьское), 16055 (river & station Naryn/Нарын), 16105 (river & station Aflatun/Афлатун)) have a gap-free overlap with the CHELSA climatology data set. Another 7 gauges (CODE 15194 (Ala-Archa River/Ала-Арча, gauge Kashka-Suu/Кашка-Суу, 15214 (Sokuluk River/Сокулук, gauge Belogorka/Белогорка), 15259 (Talas River/Талас-2,6км, gauge Uch-Koshoy/Уч Кошой), 15283 (Besh-Tash River/Беш-Таш, gauge Saz/гол.ар.Саз), 15287 (Kumush-Too River/Кюмюш-Тоо, gauge Zhany/гол.ар.Жаны), 16136 (Kurshab River/Куршаб, gauge Gulcha/Гульча), 16143 (river & gauge Changet/Чангет)) show gaps shorter than 1 year in the middle of the time series which may be imputed. The rest of the time series shows larger or multiple gaps.

## Technical Validation

Monitoring data from Central Asia is extremely rare and thus also almost impossible to validate in the proper sense of the word. We attempt to estimate the quality of the discharge data, the gauge locations and the derived watersheds for each gauge using a number of different angles and third-party data to help potential users in assessing the data quality. We use flags for each quality test performed (see Table 5 for an overview over the quality flags and their meaning).

**Table 5:** Quality flags that result from the technical validation of the data set.

Name	Description
CODE	A 5-digit number or a 10–11 digit code
qual_regime	Hydrol. regime as a number between 1 and 4. NA if no time series data is available.
qual_normq	FALSE if the average discharge deviates by more than 20% from the norm discharge.
qual_duplicates	FALSE if excessive repetitions of values occur.
qual_outliergap	Identifies time series which contain outliers with FALSE.
qual_maxmin	FALSE if max. runoff occurs immediately before or after min. runoff at monthly resolution.
qual_consistency	0 if discharge statistics are persistent over time. −1 or 1 for decreasing or increasing trends.
qual_area	FALSE if area deviates by more than 20% from literature values.
qual_module	FALSE if the specific discharge is not consistent with literature values.
qual_wb	FALSE if the norm discharge does not satisfy a simplified basin water balance.
qual_order	1, 0, or −1 for consistent, not applicable and inconsistent river runoff along a river.

### Discharge time series

No alternative *in-situ* measurements are available to validate the discharge time series data from the hydrological yearbooks. We therefore choose the following methods to quasi-validate the time series data: (a) classification of discharge time series in runoff regimes, and (b) Comparison of long-term average river runoff.

### River runoff regimes

We use the river regime classification suggested by Viktor Shultz^47^ to assign discharge regimes to the river runoff time series. If the discharge classification is not consistent with the expected classification according to basin elevation, glaciation and precipitation patterns, a $qual\_regime$ is set to FALSE, otherwise, it is set to TRUE. The classification was done in decreasing priority with the Shults coefficient *δ*, the July to September runoff in percentage of the annual runoff (${W}_{VII-IX}$), and the month of peak discharge. Where the Shults coefficient *δ* is calculated from the July to September runoff in percentage of the annual runoff (${W}_{VII-IX}$) and the March to June runoff as percentage of the annual runoff (${W}_{III-VI}$) as expressed in Eq. 1.

1 $$\delta =\frac{{W}_{VII-IX}}{{W}_{III-VI}}$$

**Fig. 6:**
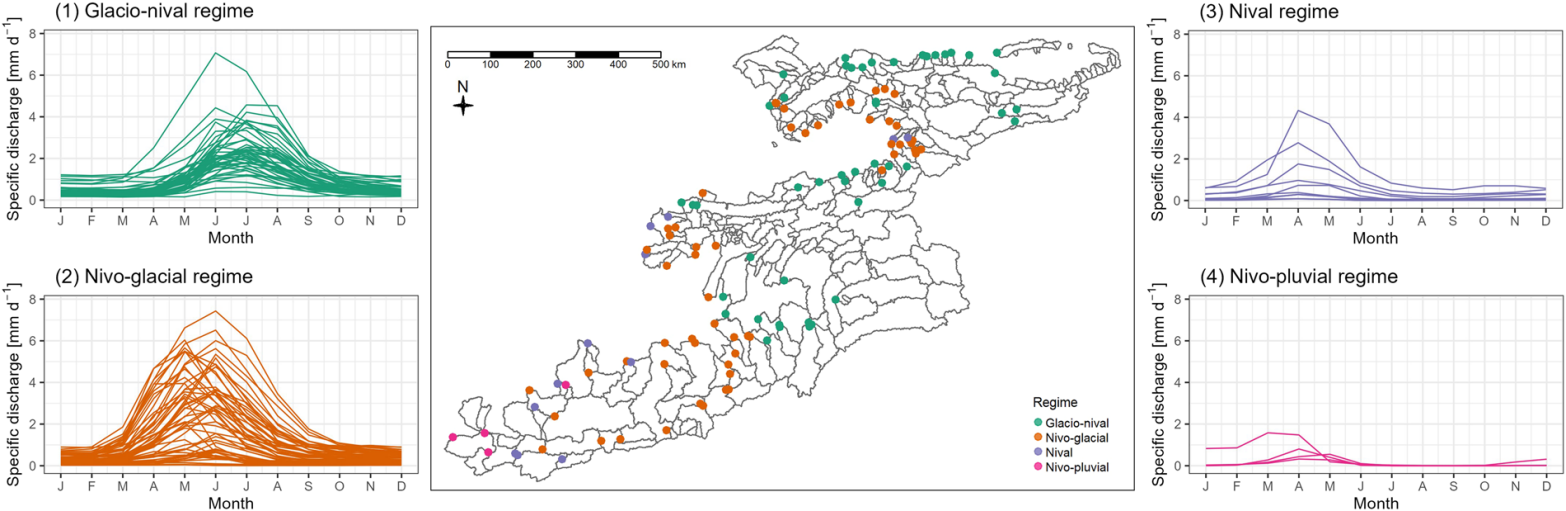
Seasonal development of specific river discharge ($discharge\_times\_series$ attribute $value$ in millimetres per day) in all gauges where time series data is available. Runoff regime classification following Schultz into glacio-nival regime, nivo-glacial regime, nival regime and nivo-pluvial regimes.

Figure 6 shows the seasonal development of discharge for 4 classes of discharge regimes according to the classification by Shultz^47^: (1) Glacio-nival regime, (2) Nivo-glacial regime, (3) Nival regime, and (4) Nivo-pluvial regime. All classified regimes are consistent with the expected regime distribution according to basin elevation, glaciation and precipitation patterns. $qual\_regime$ flags are set to the ID of the regime (1 for glacio-nival and so on). Gauges which do not have a time series or whose time series have too many missing data points to calculate discharge statistics are set $qual\_regime$ equal to NA (175 gauges). In total the data set includes 46 time series with the glacio-nival regime, 58 time series with the nivo-glacial regime, 12 time series with nival regime and 4 time series with nivo-pluvial regime.

### Comparison of average runoff

The average annual river runoff was calculated as follows: Average monthly discharge was first calculated by averaging daily data if at least 26 daily observations were available in a given month or by averaging decadal data if at least 2 out of 3 observations were available in a given month. In the second step, the annual average discharge was calculated by averaging the monthly discharge if at least 11 observations were available in a given month. Finally, the annual average discharge was calculated only if at least 8 years of data were available. As the time period, over which the norm discharge reported by the Hydromets is not known, we used the entire available time series data to calculate the average annual discharge from the time series data.

Differences in the average annual runoff between the long-term discharge provided by the Hydromets (basin attribute *q_m3s*) and the average annual runoff computed from the time series data of 20% were assumed acceptable. For three gauges, the average annual discharge deviates by more than 20% from the norm discharge reported by the Hydromets, namely gauges 8-0.000-1 M (Hari Rud River at Tir Pul), 16093 (Torkent/Торкент-То), 16137 (Kurshab River/Куршаб, gauge Kochkor-Ata/Кочкор-Ата), 16151 (Maylisu/Майлису, Kayragach/Кайрагач), 17050 (Gunt River/Гунд, Khorog/Хорог, both decadal and monthly resolution), 17223 (Sherabad River/Шерабад, gauge 0.4 km above the confluence with the Maidan River/в 0.4 км выше устья р. Майдан), and 17462 (Kysylsuu West/Кызылсу Западная, Daraut-Korgan/Дараут-Курган). These gauges are assigned quality flag *qual_normq* FALSE.

### Time series with different resolution

For two gauges, 17050 (Gunt River) and 16070 (Small Naryn River), we have time series at two different resolutions available (decadal and monthly in the case of the Gunt River and daily and decadal in the case of the Small Naryn River). Both higher and lower frequency data for the Gunt River are from the Tajik Hydromet and both higher and lower frequency data for the Small Naryn River are from the Kyrgyz Hydromet. For comparison, the daily and decadal data are aggregated to monthly data.

**Fig. 7:**
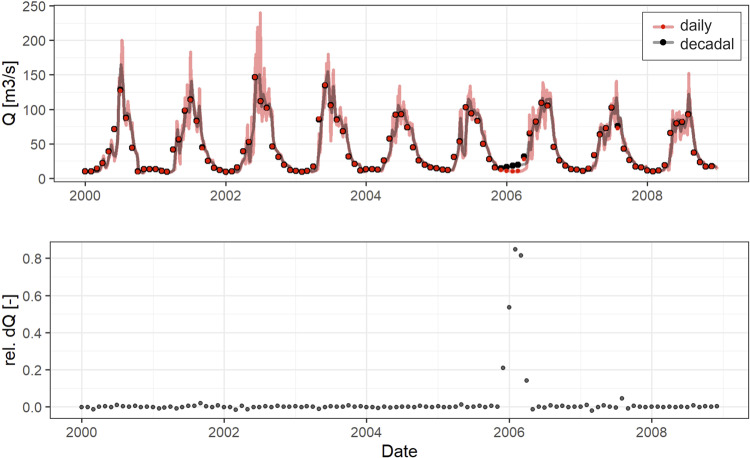
Comparison of daily and decadal discharge time series at station 16070 in the Small Naryn River. The lines represent the original time series obtained from Kyrgyz Hydromet at daily and decadal resolution (red and black respectively) and the points indicate monthly averages calculated based on the daily and decadal time series data (red and black respectively). The bottom panel shows the relative difference between the monthly aggregates of the time series with higher and lower resolutions.

Figure 7 shows the discharge time series at gauge 16070 in the Small Naryn River during the time period where both time series overlap. The daily and decadal time series are fairly consistent with a mean difference of 0.2%. One inconsistency occurs in the 2006 winter season where the decadal data shows an increase in discharge where the daily time series shows a decrease in discharge. The discrepancy between the two data sets is up to 80%. It is impossible to say which time series is correct.

**Fig. 8:**
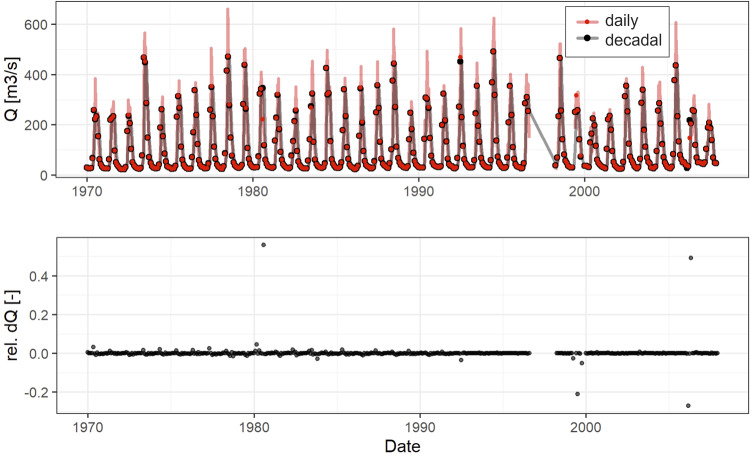
Comparison of decadal and monthly discharge time series at station 17050 in the Gunt River. The lines represent the original time series obtained from Kyrgyz Hydromet at decadal and monthly resolution (red and black respectively) and the points indicate monthly averages calculated based on the decadal and monthly time series data (red and black respectively). The bottom panel shows the relative difference between the monthly aggregates of the time series with higher and lower resolutions.

Figure 8 shows the same visualization for the Gunt River station. Also in this case, the two data sets are fairly consistent.

We fit a Normal distribution through the relative differences between the higher and lower frequency versions of the discharge time series and get standard deviations of 5% for Gunt River and 12% for Small Naryn River. These gauge stations are both important for transboundary water resources management and it is to be expected, that the monitoring process is adequate. However, errors can occur when transcribing observation values from one excel sheet to another.

### Automated time series quality tests

Inspired by Durre and colleagues^48^ we test for data integrity. All discharge values lie within reasonable boundaries, i.e. they are larger or equal to 0 and below 10’000 m^3^/s. Each time series is tested for repetitive occurrences of yearly hydrographs (no repetitions of entire years data present in the daily, decadal or monthly time series) as well as for repetitions of monthly data (no repetitions of entire months data present in the daily or decadal time series).

We further flag gauges with time series that include repetitions of discharge values with *qual_duplicates* FALSE. We thereby look for non-zero repetitions of at least 40 daily values for time series at daily resolution, 9 decadal values for time series at decadal resolution, or 3 monthly values for time series at monthly resolution. The 27 gauges flagged with *qual_duplicates* FALSE are 16068 (Big Naryn), 15212 (Ak-Suu-s./р.Ак-Суу-с., Chon-Aryk/Чон-Арык), 15285 (Ur-Maral/р.Ур-Марал, October/Октябрьское), 15287 (Kumush-Too/р.Кюмюш-Тоо, Zhany/гол.ар.Жаны), 16105 (Aflatun/Афлатун), 16133 (Kulduk/Кульдук, Cary-Bulak/Сары-Булак), 16134 (Donguztau/Донгузтау), 16153 (Akbura/Акбура), 16193(Gavasay/Акбура, Gava/Гава), 16198 (Sokh/Сох, Sarykanda/Сарыканда), 16202 (Chadak/Чадак, Djulaysay/Джулайсай), 14-1.R00-2A (Taloqan River at Pul-i-Chugha), 8-0.000-1 M (Hari Rud River at Tir Pul), 8-0.000-3 S (Hari Rud at Pul-i-Pashtoon), 8-1.R00-9T (Senjab River at Khush Rabat), 10-1.1L0-7A (Qaisar Gauge), 9-1.000-1 A (Char Takhta Gauge), 9-2.000-1 A (Chil Dukhtaran Gauge), 9-5.L00-1A (Luka-I-Surkh Gauge), and 16390 (Sayram/Р.Сайрам, Tasarik/с.Тасарык). Reasons for such duplicates include reporting errors, a dry riverbed or frozen riverbed, etc..

We test for outliers by calculating the gap size in the histogram of the daily, decadal and monthly time series^48^. None of the gaps are larger than 100 m^3^/s for daily, decadal or monthly time series except for gauge 14-0.000-4 M (Kunduz River at Baghlan) which is flagged with $qual\_outliergap$ FALSE.

In typical snow-melt regimes, peak discharge does not immediately follow minimum discharge in seasonal hydrographs. We calculate the time difference between maximum and minimum annual discharge in complete years and flag gauges which have at least one occurrence of maximum discharge immediately following or preceding minimum discharge with $qual\_maxmin$. Four stations are flagged with $qual\_maxmin$ FALSE, namely 14-0.000-6 M (Kunduz River at Pul-i-Konda Sang, 14-9.5R0-1T (Foladi River at Bamyan), 13-0.000-2 M (Sayad Gauge), and 16175 (Kokcu - Mouth).

To test if the average monthly and annual discharge is consistent over time, we calculate mean monthly and mean annual discharge as well as standard deviations of monthly and annual discharge in up to 7 30-year periods, depending on the length of the time series: from 1940 to 1970, 1950 to 1980, 1960 to 1990, 1970 to 2000, 1980 to 2010, and from 1990 to 2020. We only calculate the statistics if more than 26 daily or more than 2 decadal values are available per month, if at least 11 monthly values are available per year and if at least 8 years of data are available within a 30-year period. If the mean plus/minus one standard deviation ranges of the monthly or annual discharge values across the 7 averaging periods do not overlap, we detect a strong change in mean monthly and annual discharge respectively. The flag $qual\_consistency$ is assigned −1 when an overall decrease of average monthly and annual discharge is detected, 0 when no change is detected and +1 when an overall increase in discharge is detected. Gauges with no discharge time series information or gaps in the time series are assigned $qual\_consistency$ NA. Two time series display strong decreasing discharge trends, namely 15102 (Chu/Чу, Kochkorka/Кочкорка) and 15215 (Kara-Balta - Sosnovka/Кара-Балта-Сосновка). Both time series show a distinct increase in flows in the winter half-year.

### Gauge locations and basin outlines

**Fig. 9:**
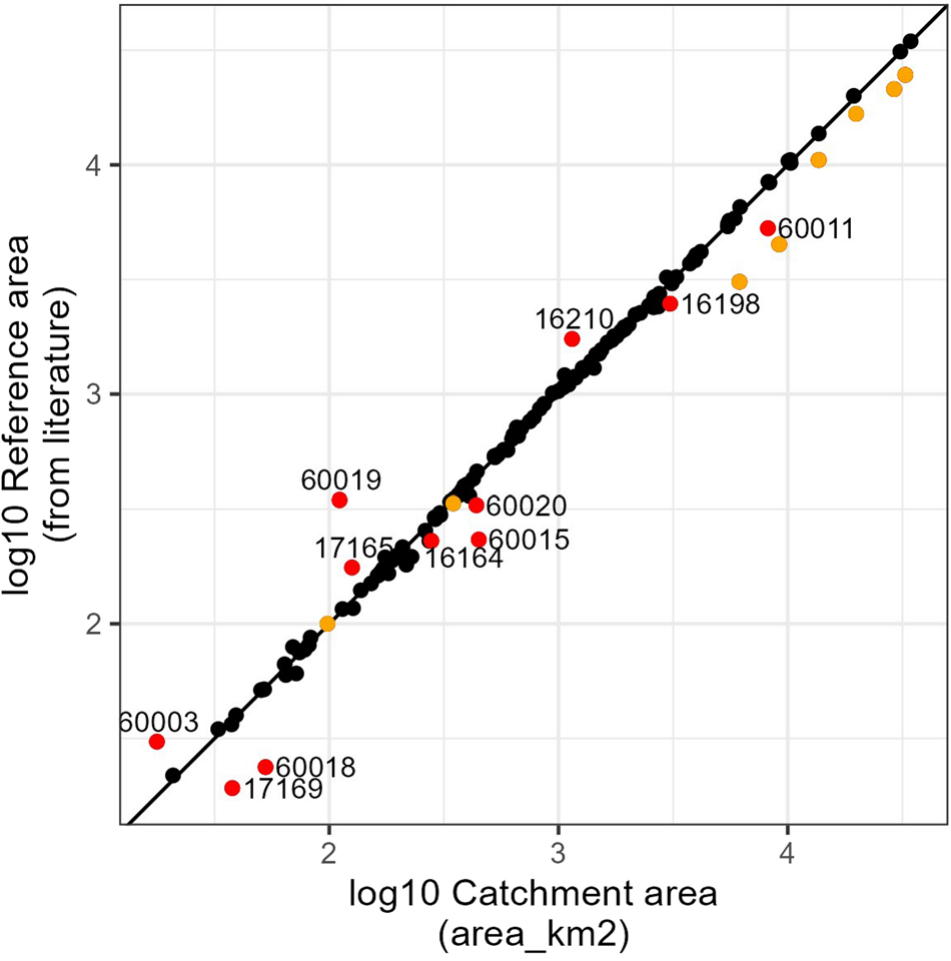
Area derived from the catchment delineation ($area\_km2$) vs. the basin areas from literature (Reference area)^16–18^. Orange points indicate basins extending to the far east parts of the Pamir mountains where the basin areas from the literature are deemed inexact. Red points with gauge labels indicate a deviation of more than 20% between literature areas and derived areas.

The gauge locations and basin outlines have been validated visually in QGIS by zooming in on selected gauges and basins and following the basin outline on a topographical map (DEM^14^ and hillshades derived on DEM with a glacier area overlay^43^. It was thus visually ensured that the basin boundaries follow the watershed boundaries. It was further verified that the gauge attributes $NORTHING$, $EASTING$, $LON$, and $LAT$ lie within the bounding box of the basin polygons. The basin areas calculated on the catchment outlines (basin attribute $area\_km2$) have been compared to 138 basin areas found in the literature^16–18^ (see Fig. 9). The basin areas in the far east of the Pamirs (CODEs 17057 to 17065, orange points in Fig. 9) may not be correct in the literature as the Soviet topographical maps only extended up to the Chinese border and the catchment delineation may not have been correct then. These basins are thus excluded from the comparison with catchment areas from the literature. Figure 9 shows catchments of gauges with a deviation of at least 20% from the area reported in the literature in red with the gauge CODEs as labels. The placement of these gauges was re-checked with the available literature but the catchments of the gauges could not be improved based on the available information and the source of this deviation in the area is unknown. The authors did not wish to remove these gauge points because some of them may be of public interest (e.g. downstream of a large mining pit). The 11 gauge catchments in question (CODE 60003 (Baydula/Байдула), 60011 (Kekemeren/Кекемерен, 1.8 km Djumgol), 60015 (Kumtor/Кумтор, Tyan-Shan/Тян), 60018 (Nichkesay/Ничкесай), 60019 (Orto/Орто, Kugandy/Куган), 60020 (Ottuk/Оттук), 16164 (Abshirsay/Абширсай, Uch-Terek/Уч-Терек), 16198 (Sokh/Сох, Sarykanda/Сарыканда), 16210 (Khodjabakirgan/Ходжабакирган, Andarkhan/Андархан), 17165 (Siama/Сиама, Igizak Mouth/Устье Игизак), and 17169 (Kurortnaya/Курортная, Kusheri/Хушъери)) are, however, excluded from the discussion of the basin attributes. Only for gauge 16198 discharge time series are available. These 11 basins are assigned flag $flag\_area$ FALSE. Possible explanations for area discrepancies may be incorrect placing of gauge location, copying error in the present data set or in the literature, or incorrect delineation of the basin area because of problems in the underlying DEM.

### Norm discharge

While many Hydromets have very stringent monitoring quality requirements (errors of less than 5% in discharge), the actual monitoring process by the gauge operators is often less than optimal as anecdotal evidence suggests. Errors in monitoring have for example occurred by the observer moving the gauge location closer to his hut or by observers inventing water level data. The cross-section at the gauge location may not be maintained properly and the accumulation (or erosion) of sediments in the cross-section can lead to water level readings which are not consistent with the water-level discharge relationship and thus lead to an over- (or under-) estimation of the river runoff. We, therefore, assume an error in the discharge of 20% which is a widely accepted rule-of-thump value for errors in river discharge among hydrologists. *In-situ* measured validation data for the norm discharge does not exist. Therefore, we use different methods using third-party data to assess the quality of the norm discharge data.

### Test discharge-elevation relationship

**Fig. 10:**
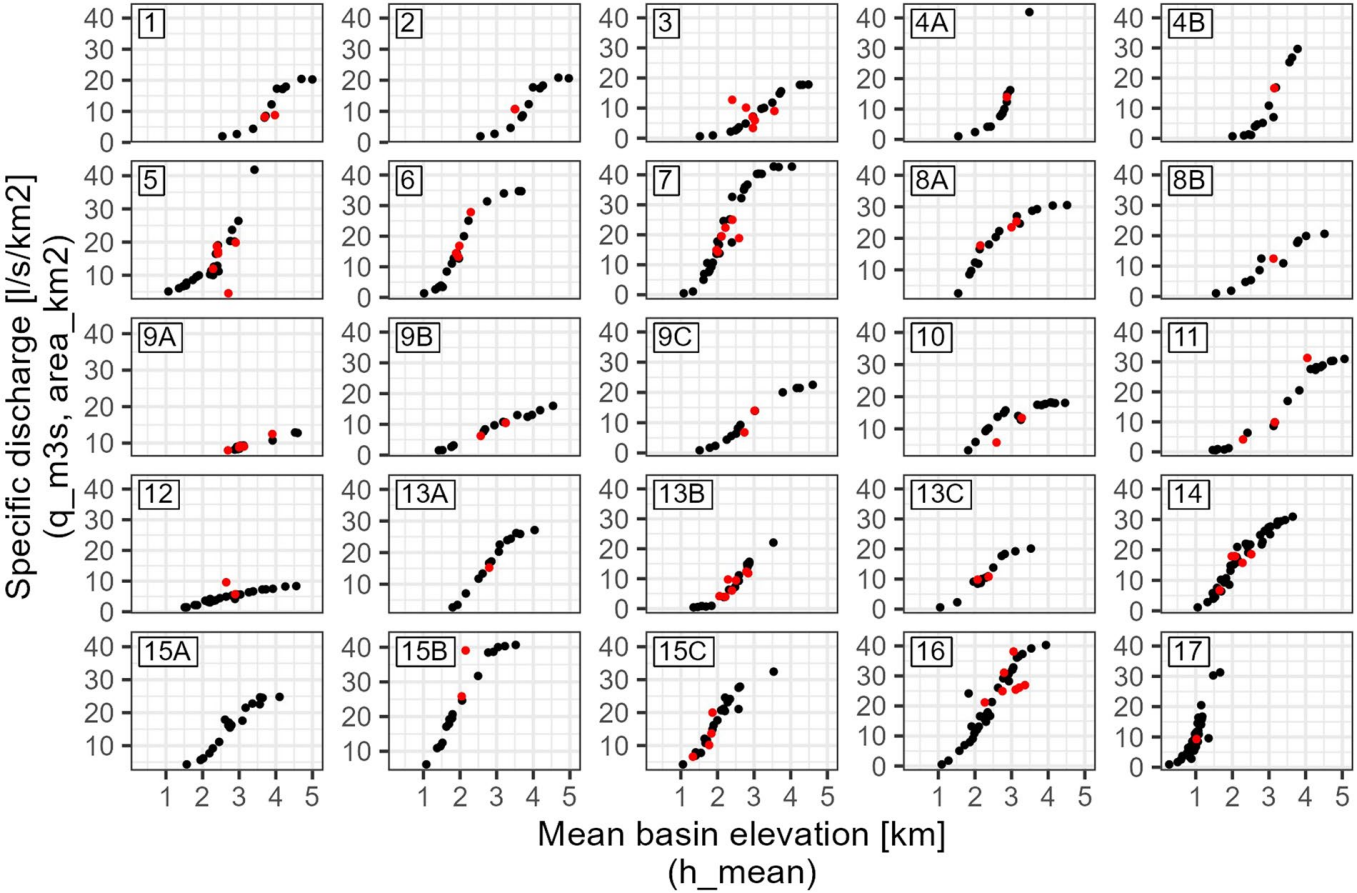
Specific discharge vs. mean basin elevation as digitized from Surface Water Resources, Vol. 14, Issue 1^16^ (black) and calculated from the present data set (red). The oro-hydrographic regions are: 1 - Big Naryn basin, 2 - Small Naryn basin, 3 - Middle part of Naryn basin, 4 A - Upper part and right side of the middle part of Kekemeren basin, 4B - Left side of Kekemeren basin and Western Karakol basin, 5 - Low part of Naryn basin, 6 - Karasu Right basin, 7 - Northern part of the south-western slope of Ferghana Range, including right side tributaries of Yassy River, 8 A - Yassy and Karakuldzha basins, 8B - Tar basin, 9 A - Kurshab and Akbura basins, 9B - Abshirsay and Isfayramsay basins, 9 C - Shakhimardan basin, 10 - Aravan basin, 11 - Western part of the northern slope of Alay Range and eastern part of the northern slope of Turkestan Range, 12 - Western part of the northern slope of Turkestan Range, 13 A - Padsha-Ata basin, 13B - Kassansay and Gavasay basins, 13 C - Western part of the south-eastern slope of Kuramin Range, 14 - Akhangaran basin, 15 A - Upper and middle part of Chatkal basin, 15B - Right side of muddle part of Chirchik basin, 15 C - Left side of muddle part of Chirchik basin, 16 - Upper part of Arys basin and Pskem basin, 17 - Rivers of south-western slopes of Karatau and Boralday-Tau Ranges.

Figure 10 shows manually digitized observations of specific discharge and mean basin elevation^16^ for 17 oro-hydrographic regions in the Syr Darya basin in black and data from the present data set in red. The correspondence of the norm discharge with the historical data is satisfying for most gauges. Outliers are visible in oro-hydrographic zone 3 which is described as the middle part of the Naryn basin (i.e. from the confluence of the small and big Naryn rivers to the confluence of the rivers Naryn and Kekemeren. Gauges which do not satisfy the relationships are flagged $qual\_module$ FALSE. Reasons for discrepancies between literature data and the present data can be reporting errors, errors in gauge location or catchment area delineation, but also natural variability of discharge or changes in discharge since the collection of the literature data more than 50 years ago. For example, the tributary basins in the large middle Naryn oro-hydrographic region are highly heterogeneous and variable in their discharge production. Outliers in the discharge-elevation relationship in Fig. 10 in the middle Naryn basin are therefore not necessarily wrong but may reflect natural variability within the region. As the exact gauge locations which were used to derive the literature relationship are not documented, it is further possible that gauges flagged here were not used to derive the relationships. The flagged gauges are 16081 (Kekirim-Kara-Tabylga/Кекирим-Ка), 16298 (Nauvalisoy/Наувалысай, Sidjak/Сиджак), 60013 (Kekemeren/Кекемерен, Sarykamysh), 60018 (Nichkesay/Ничкесай), 60038 (Oygaing/Ойгаинг, above mouth Koksu, выше устья р. Коксу), and 60039 (Koksu - mouth/Коксу - устье).

### Water balance

The long-term water balance over a basin, neglecting sub-surface flows, is given as $Q=P-E$ where *Q* is the long-term norm discharge at the outlet of the basin, *P* is the long-term norm precipitation over the basin area, and *E* is the long-term norm actual evaporation over the basin area. Since global warming is accelerating glacier mass loss which contributes to a de-storage of water in glaciated basins, the water balance can be extended to include glacier mass loss *dS* as $Q=P-E+dS$ (neglecting other potential long-term changes of storage in the basin such as permafrost).

As mentioned above, the time period to calculate the norm discharge reported by the Hydromets (attribute $q{\rm{\_}}m3s$) is not available. It is therefore not possible to choose weather data from the exact same time period as the discharge measurement period. Also, publicly available weather station data does not cover the remote mountainous catchments. We thus have to rely on publicly available gridded data products. We calculate average annual precipitation for each basin as mean over 7 gridded precipitation products (${P}_{mean}$): CHELSA v2.1 (${P}_{CHELSA}$), WorldClim v21^32^, CHPclim^34^, CRU^49^, GPM IMERG^50^, CHIRPS^51^, and APHRODITE^52^ which all have shown acceptable performance in Central Asia^53–57^. Depending on the basin, the difference in precipitation can be up to 1'000 mm/a from one precipitation product to another. To include the uncertainty in precipitation, we calculate the standard error ${s}_{P}=\sqrt{\frac{1}{N-1}{\sum }_{i=1}^{N}{({P}_{i}-{P}_{mean})}^{2}}/\sqrt{n}$ over the *N* = 7 different precipitation products for each basin. This standard error was also assumed to hold for the mean over all precipitation products as well as for individual precipitation products. All precipitation products are extracted between 1981 and 2010 except for GPM IMERG which is only available after 2000 and was extracted between 2000 and 2010.

Actual evaporation was estimated using two different methods: (a) Based on the aridity index and precipitation from CGIAR/WorldClim^32,35^ and CHELSA respectively using the Budyko framework^58^ with Fu’s equation as done by Beck and colleagues^23^ whereby the mean actual evaporation using these methods is denoted as ${E}_{Budyko}$ and (b) using gridded data products SSEBop^59^ and PML^60^ which both show good performance on global scale^61^ (the mean actual evaporation from SSEBop and PML is denoted as ${E}_{grid}$). Where the mean over all 4 data products & methods has been used we write ${E}_{mean}$. Actual evaporation estimated using the Budyko framework was consistently higher than the average between SSEBop and PML (by 170 mm on average). SSEBop data is available between 2003 and 2020, PML data is available between 2001 and 2020. We assume that the standard error is a suitable error statistic to represent the uncertainty of the average of actual evaporation from either the Budyko method or from the gridded data sets thus resulting in ${s}_{E,Budyko}$ and ${s}_{E,grid}$ calculated analogous to ${s}_{P}$ with $N=2$ and to ${s}_{E}$ calculated with $N=4$.

Glacier mass loss from Hugonnet and colleagues (denoted as *dS*) and their error estimates (denoted as ${s}_{dS}$) were used^45^.

We have compared 6 varieties of water balance equations for *Q*, namely ${P}_{CHELSA}-{E}_{Budyko}+dS$, ${P}_{CHELSA}-{E}_{grid}+dS$, ${P}_{mean}-{E}_{mean}+dS$, ${P}_{mean}-{E}_{grid}+dS$, ${P}_{mean}-{E}_{grid}$, ${P}_{CHELSA}-{E}_{grid}$, whereby ${P}_{CHELSA}$ refers to $bio12$, ${P}_{mean}$ is the mean over 7 precipitation products as described above, ${E}_{Budyko}$ is the average actual evaporation estimated using the Budyko framework, and ${E}_{grid}$ is average actual evaporation from SSEBop and PML.

The errors were propagated accordingly by summing up the standard errors of the respective variables in each equation. For the first equation this yields ${s}_{P}+{s}_{E,Budyko}+{s}_{dS}$ as an example.

We compared typical model performance statistics used in hydrology (mean error, mean absolute error, root mean squared error (RMSE), percent bias, Nash-Sutcliffe efficiency, index of agreement, Klinge-Gupta efficiency, and volumetric efficiency among others) using the R package hydroGOF^62^. The water balance model ${P}_{CHELSA}-{E}_{grid}$ thereby showed the best performance with an RMSE of 91 m^3^/s and volumetric efficiency of 0.5. The associated uncertainty of the runoff estimated with the water balance is then the sum of the standard errors over the 7 precipitation products and the 2 gridded evaporation products (${s}_{P}+{s}_{E,grid}$).

**Fig. 11:**
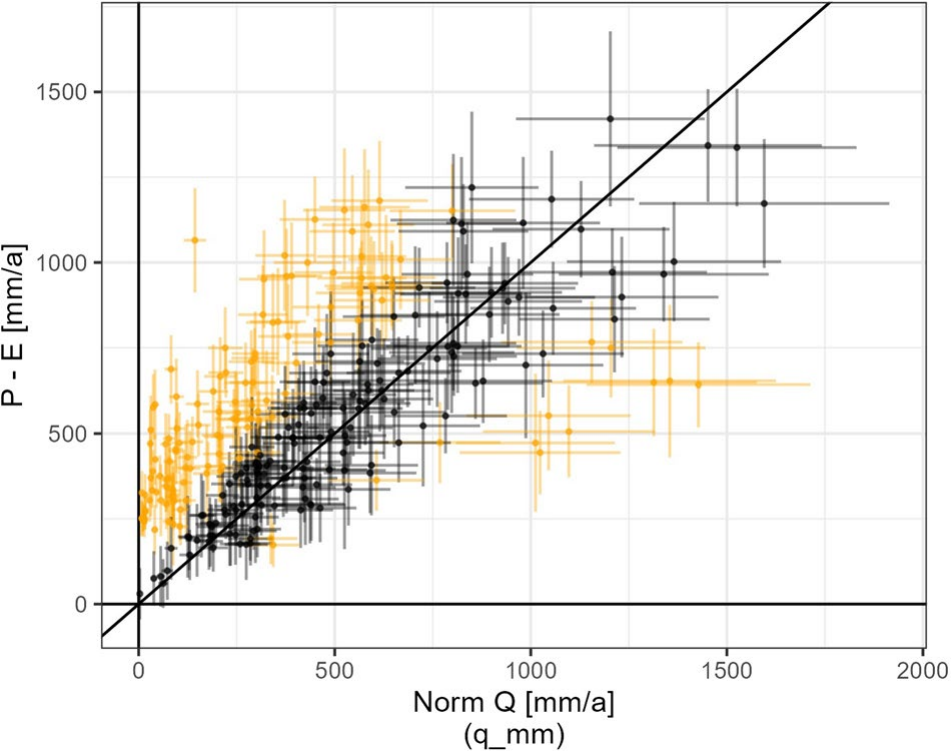
Scatter plot of norm discharge (attribute $q\_mm$) vs. ${Q}_{estimated}={P}_{CHELSA}-{E}_{grid}$. The crosses indicate the uncertainties in norm discharge in the horizontal dimension and in ${P}_{CHELSA}-{E}_{grid}$ in the vertical dimension. Orange crosses indicate basins for which the water balance is deemed not fulfilled within given uncertainties (quality flag $qual\_wb$ FALSE).

Figure 11 shows the norm discharge (${q}_{mm}$) against the discharge calculated using the water balance approach described in the paragraph above with associated uncertainties. Gauges for which there is no overlap between the discharge calculated from the water balance plus/minus the combined uncertainty of the calculated discharge and the norm discharge plus/minus the uncertainty of the norm discharge (assumed to be 20%), are assigned quality flag $qual\_wb$ FALSE (this is the case for 46% of the gauges). The formula used to assess the overlap and to accept the water balance is $({P}_{CHELSA}-{E}_{grid})+({s}_{P}+{s}_{E,grid}) < {q}_{mm}\cdot (1-0.2)$ & $({P}_{CHELSA}-{E}_{grid})-({s}_{P}+{s}_{E,grid}) > {q}_{mm}\cdot (1+0.2)$. It should be noted that the choice of the error statistic used for estimating the uncertainty of a flux heavily influences the result, as does the choice of the precipitation and evaporation products.

### Gauge network

**Fig. 12:**
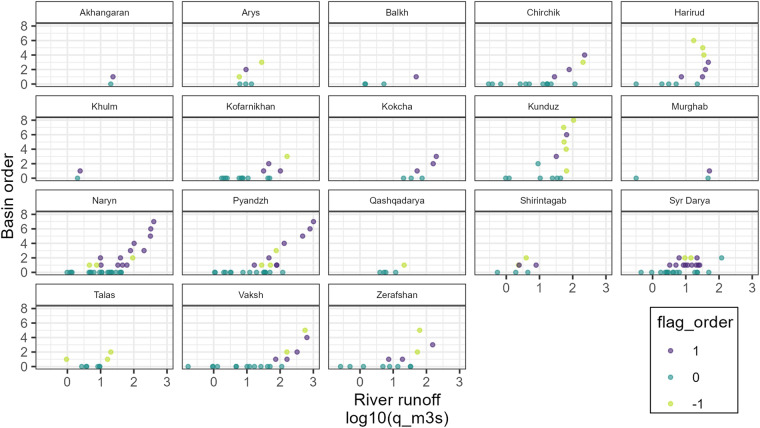
Basin discharge generally increases with increasing basin order within a basin. Exceptions are for example the Harirud River basin (top right tile) where downstream discharge decreases (probably due to abstractions). flag_order 0 indicates headwater basins, flag_order 1 indicates downstream gauges with discharge larger or equal to the sum of the discharge of upstream gauges and flag_order -1 indicates gauges with downstream gauges discharge smaller than the sum of the discharge of upstream gauges.

To validate the mapping of the gauge network, the basin order was visualised against norm discharge for each gauge that passed the area validation (Fig. 12). Unless there is an abstraction of river water, downstream gauges should show higher runoff than upstream gauges. This is given in the Naryn basin for example. In the Harirud basin, river runoff decreases in the higher-order downstream gauges. An inspection of an optical satellite image shows irrigated agriculture along the lower reaches of the Harirud river, accounting for the decrease in river discharge despite increasing river order. We flag downstream gauges which have lower discharge than the sum of upstream gauges at the same basin order with $qual\_order$ −1. Gauges with discharge larger or equal to the sum of the discharge of upstream gauges at the same basin order are assigned $qual\_order$ 1. Gauges for which this test does not apply, i.e. headwater gauges, are assigned $qual\_order$ 0.

### Time series of basin attributes

**Fig. 13:**
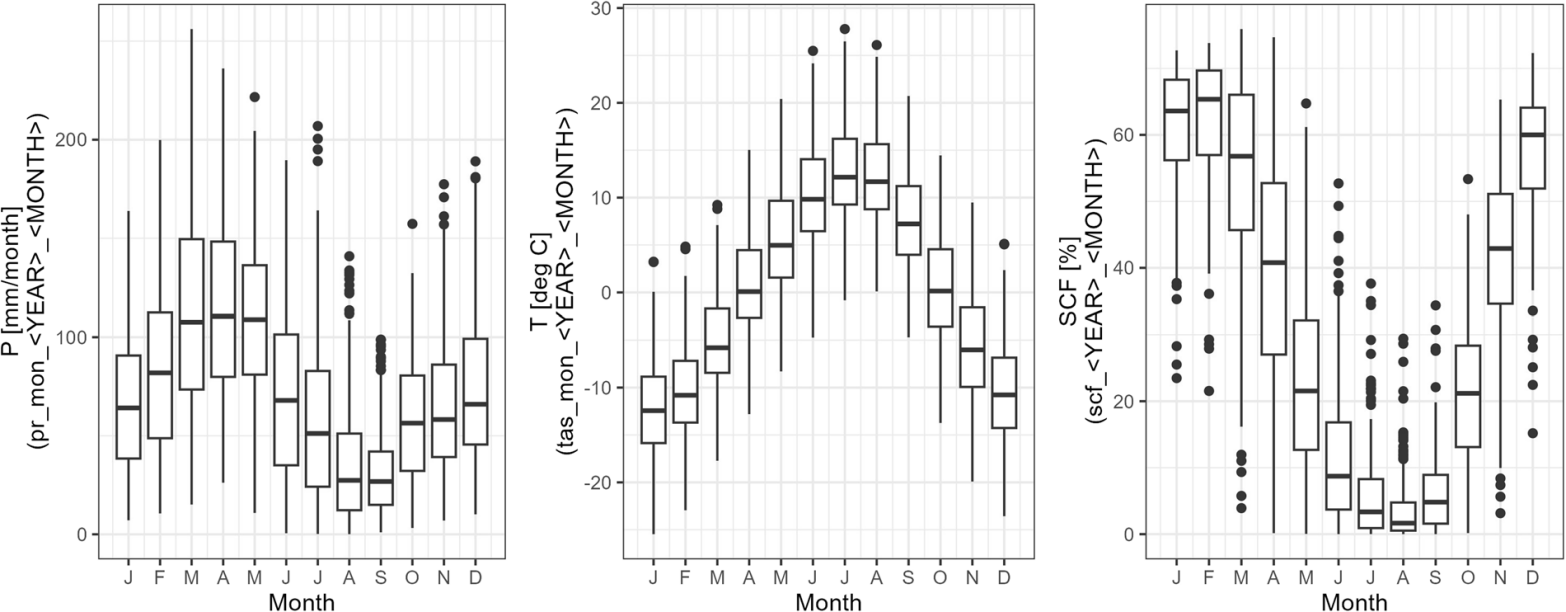
Seasonal development of precipitation (P), temperature (T), and snow-covered fraction (SCF) over all basins. The figures are derived from the basin attributes $pr\_ann\_ < YEAR > \_ < MONTH >$, $tas\_ann\_ < YEAR > \_ < MONTH >$, and $scf\_ < YEAR > \_ < MONTH >$ respectively.

From the time series of basin average precipitation, temperature, and snow-covered fraction the average seasonal pattern over all basins was derived (see Fig. 13).

The basins show highly variable precipitation patterns. The summer months are dry in all basins with an average of below 50 mm/month of precipitation. Precipitation falls mostly in spring and winter with averages of 70 to 120 mm/month. The temperature pattern is more homogeneous with maximum temperatures in the summer of 12 degrees Celsius (deg. C) on average and minimum temperatures in the winter months of −2 deg. C on average. The snow-covered fraction is highly variable as well but shows the expected pattern of a high snow-covered fraction in winter months and a low snow-covered fraction in summer months.

**Fig. 14:**
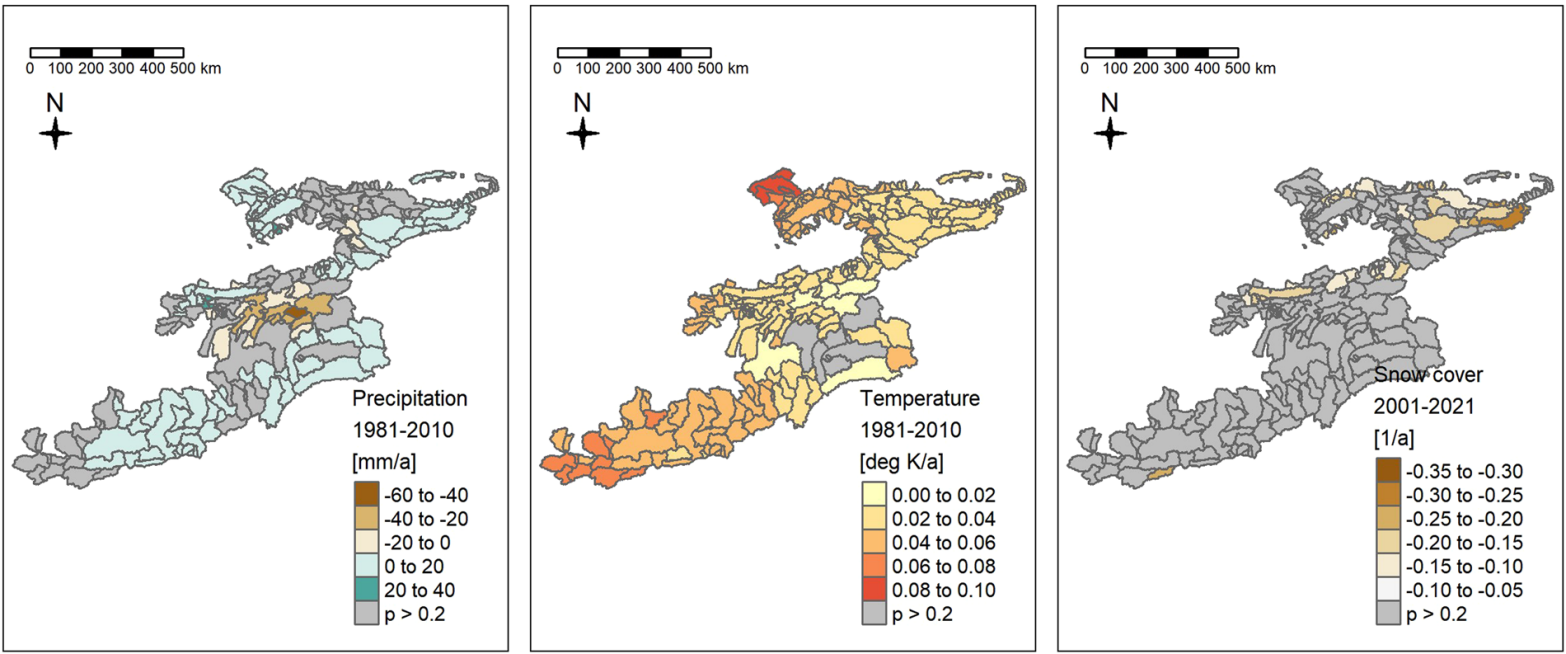
Sen’s slopes over 30 years of monthly precipitation (left) and temperature (center) and snow cover fraction (right) time series. We chose an arbitrary cutoff value of 0.2 for the p.value for visualizing Sen’s slopes. Please note that precipitation and temperature time series are extracted for the period between 1981 and 2010 from CHELSA v2.1 while snow cover fraction is extracted from MODIS for the period between 2001 and 2021.

**Fig. 15:**
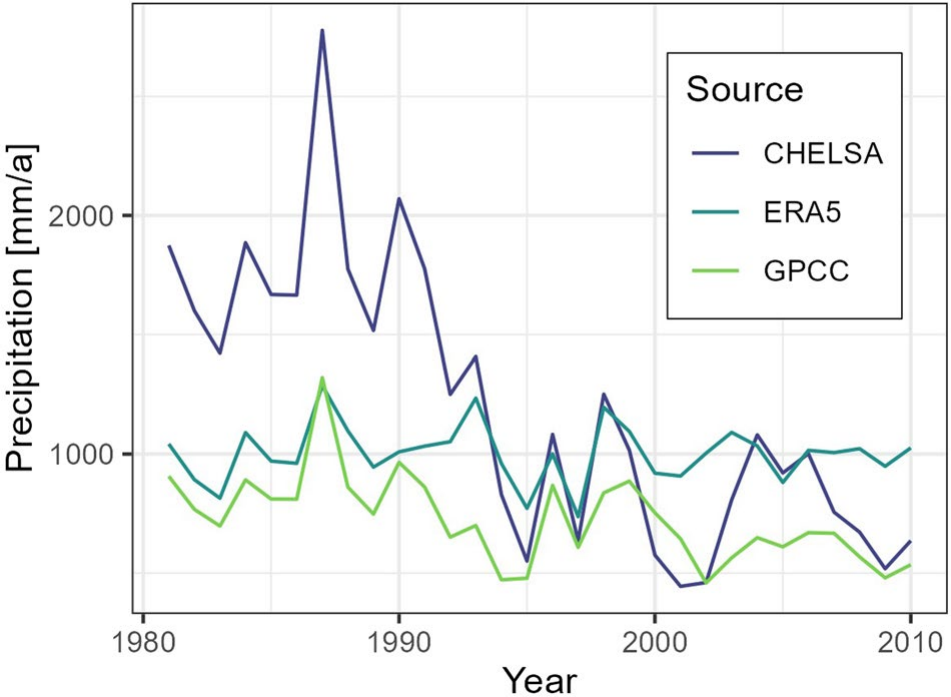
Average annual basin precipitation of gauge 17107 (mean elevation of 4011 masl, extracted from CHELSA v2.1^22^, ERA5^69^ and GPCC^64^. The significant decrease of CHELSA precipitation in the high-altitude basin can be explained by the decrease of precipitation in the GPCC precipitation product. The ERA5 precipitation does not show a step-change in precipitation in this basin.

We calculate Sen’s slopes^63^ on annual aggregates of the monthly time series of average precipitation per basin, average temperature per basin (both CHELSA v2.1) and average snow cover fraction per basin (extracted from MODIS) to check for trends in the data sets. For Fig. 14 we chose a threshold of 0.2 for the p-value meaning that we accept a 1 in 5 chance of displaying a spurious trend. About 40% of the Sen’s slopes calculated on annual precipitation sums have a p-value above 0.2 and are thus not visualized in Fig. 14. Precipitation between 1981 and 2010 shows strong trends in the southern Alai mountains of −40 to −60 mm/a or −1200 to −1800 mm in 30 years (for example basin of gauge 17107). This basin and the surrounding ones show a step decline of annual precipitation of around 1'000 mm in the mid 90ies (see Fig. 15). This step-change in the CHELSA precipitation originates from the GPCC data set which was used for bias correction^21^. The station density of the GPCC data in Central Asia reduced considerably following the demise of the Soviet Union^64^. A cursory comparison of gridded precipitation products in Central Asia that are corrected with station data suffer from the same problem of changes in station density. As long as such problems persist in precipitation products, we suggest applying a weighted ensemble approach, using several precipitation products for hydrological modelling in Central Asia.

CHELSA temperature trends in the basins look reasonable at a first glance albeit high (for example up to 0.1 deg. K/a or 3 deg. K over 30 years in the Arys basin in the north of the study area). While spatial temperature distributions are less variable than precipitation distributions and thus also the problem of station density reduction may be less prominent, it is advisable to evaluate the several temperature products prior to hydrological modelling.

Trends in snow cover fraction (right tile in Fig. 14) are less pronounced than trends in temperatures or precipitation. They do however show a marked decrease in snow cover in the eastern Tien Shan mountains (basin of the Naryn) and the Zarafshan River basin.

### Comparison with discharge from the global runoff data centre

The Global Runoff Data Centre (GRDC) (https://www.bafg.de/GRDC/EN/01_GRDC/grdc_node.html) provides daily or monthly discharge time series of stations all over the globe. To compare the CA-discharge data set with the GRDC data set (hereafter abbreviated with GRDC), we manually mapped the gauge CODE to each gauge location in QGIS. 84 gauges from GRDC were identified to overlap with gauges in the CA-discharge data set, whereby 12 locations in the GRDC data set were found to be wrongly or imprecisely geolocated. In the mountainous area of Central Asia, the CA-discharge data has time series from 52 more gauges available. The CA-discharge data set further features longer time series. However, for the Afghan stations, GRDC has daily values where CA-discharge has monthly values only.

### Concluding remarks on data validity

Without *in-situ* measurements of river runoff, the possibilities to validate the time series data are limited. The same goes for gauge locations. Future data users need to be aware of these limitations. We are, nevertheless, convinced that the open-access publication of this as-good-as-it-gets data set is a highly valuable contribution to the hydrology community.

## Usage Notes

The data set is named CA-discharge and stored as a geopackage with point features, polygon features, and attribute tables. The geopackage can be downloaded from Zenodo^7^. The geopackage can be opened in a GIS software like QGIS or in a scripting language like R or Python.

### Instructions for R

Open R and navigate to the location where you have stored the geopackage CA-discharge.gpkg. To view the content of the geopackage type sf::st_layers(“CA-discharge.gpkg”). This will print a table similar to Table 1 and provide you with the names of the layers in the geopackage. To read the content of individual layers, adapt the following command to read in the gauges layer: gauges < - sf::st_read(“CA-discharge.gpkg”, layer = “gauges”). In your workspace, you will now have an object gauges of class sf containing 299 features (rows) and 20 fields (columns) as described in Table 2.

The R-scripts that were used to generate the code are available from Zenodo^7^. Users wishing to reproduce workflow using the provided scripts, require a working knowledge of R. A README guides through the process.

### Instructions for QGIS

Store the geopackage in a convenient location. In your QGIS browser window, navigate to the file CA-discharge.gpkg. With a double-click on the geopackage, the list of layers is opened. Load a layer to your map with a double-click.

## Data Availability

The data set is available from Zenodo^7^. R scripts used to pre-process the data is available from the same Zenodo repository^7^. The GEE code for the extraction of snow cover faction is available from Zenodo^65^.

## References

[1] Siegfried T (2012). Will Climate Change Exacerbate Water Stress in Central Asia?. Climate Change.

[2] Yapiyev V, Sagintayev Z, Inglezakis VJ, Samarkhanov K, Verhoef A (2017). Essentials of Endorheic Basins and Lakes: A Review in the Context of Current and Future Water Resource Management and Mitigation Activities in Central Asia. Water.

[3] Apel H (2018). Statistical forecast of seasonal discharge in Central Asia using observational records: Development of a generic linear modelling tool for operational water resource management. Hydrology and Earth System Sciences.

[4] Barandun, M. *et al*. The state and future of the cryosphere in Central Asia. *Water Security* **11**, 10.1016/j.wasec.2020.100072 (2020).

[5] Gerlitz L, Vorogushyn S, Gafurov A (2020). Climate informed seasonal forecast of water availability in Central Asia: State-of-the-art and decision making context. Water Security.

[6] Barbarossa V (2018). FLO1K, global maps of mean, maximum and minimum annual streamflow at 1 km resolution from 1960 through 2015. Scientific Data.

[7] Marti B (2023). Zenodo.

[8] Kazakhhydromet. Kazakh hydrological yearbooks. Address: Address: National hydrometeorological service of Kazakhstan (Kazhydromet) (Национальная гидрометеорологическая служба Казахстана), 11/1 Mangilik El avenue, Astana, Republic of Kazakhstan, 010000 (2022).

[9] Kyrgyzhydromet. Kyrgyz hydrological yearbooks. Address: The Agency on Hydrometeorology Under The Ministry Of Emergency Situation Of The Kyrgyz Republic (Гидрометеорологическая служба при Министерстве чрезвычайных ситуаций Кыргызской Республики,, 13/1 Kerimbekov, Bishkek Kyrgyzstan (2022).

[10] Tajikhydromet. Tajik hydrological yearbooks. Address: Agency for Hydrometeorology of Tajikistan, Bobjon Gafurov 373 street, Dushanbe, Tajikistan (2022).

[11] Uzbekhydromet. Uzbek hydrological yearbooks. Address: Center for hydrometeorological services of the Republic of Uzbekistan (UZHYDROMET) (Центр гидрометеорологической службы Республики Узбекистан (УЗГИДРОМЕТ)), 1-й,, Bodomzor Yuli Street, Tashkent, Uzbekistan (2022).

[12] Moritz S, Bartz-Beielstein T (2017). imputeTS: Time Series Missing Value Imputation in R. The R Journal.

[13] Lindsay JB (2016). Whitebox GAT: A case study in geomorphometric analysis. Computers and Geosciences.

[14] NASA JPL. NASA Shuttle Radar Topography Mission Global 1 arc second [Data set]. 10.5067/MEaSUREs/SRTM/SRTMGL1.003 (2013).

[15] Pebesma E (2018). Simple Features for R: Standardized Support for Spatial Vector Data. The R Journal.

[16] Main Directorate of the Hydrometeorological Service under the Council of Ministers of the USSR (original: Главное управление гидрометеорологической службы при совете министров СССР) (ed.) Surface Water Resources of the USSR, vol. 14 Central Asia, issue 1 Syr Darya river basin (original: Ресурсы Поверхностных Вод СССР, том 14 Средняя Азия, выпуск 1 Бассеин р. Сырдарьи) (Hydrometeorological Publishing House (original: Гидрометеорологическое Издательство), Leningrad, 1969). URL http://www.cawater-info.net/library/rus/hist/resources-syrdarya/pages/001.htm.

[17] Main Directorate of the Hydrometeorological Service under the Council of Ministers of the USSR (original: Главное управление гидрометеорологической службы при совете министров СССР) (ed.) Surface Water Resources of the USSR, vol. 14 Central Asia, issue 3 Amu Darya river basin (original: Рессурсы Поверхностных Вод СССР, том 14 Средняя Азия, выпуск 3 Бассеин р. Амударьи) (Hydrometeorological Publishing House (original: Гидрометеорологическое Издательство), Leningrad, 1969). URL http://www.cawater-info.net/library/rus/hist/resources-amudarya/pages/001.htm.

[18] Ivanov, Y. N. Research report - Develop an objective method for assessing of the water resources of rivers carrying water to Uzbekistan (intermediate) (original: Отчет о научно-исследовательской работе – Разработать объективный метод оценки водных ресурсов рек, несущих воду в Узбекистан (промежуточный)). Tech. Rep., Uzbek Hydrometeorological Research Institute (original: Научно-исследовательский Гидрометеорологический Институт) (2010).

[19] Horn B (1981). Hill shading and the reflectance map. Proceedings of the IEEE.

[20] Wilson MFJ, O’Connell B, Brown C, Guinan JC, Grehan AJ (2007). Multiscale Terrain Analysis of Multibeam Bathymetry Data for Habitat Mapping on the Continental Slope. Marine Geodesy.

[21] Karger DN (2017). Climatologies at high resolution for the earth’s land surface areas. Scientific Data.

[22] Karger, D. N. *et al*. Climatologies at high resolution for the earth’s land surface areas, 10.16904/envidat.228 (2021). 10.1038/sdata.2017.122 PMC5584396 28872642

[23] Beck HE (2020). Bias Correction of Global High-Resolution Precipitation Climatologies Using Streamflow Observations from 9372 Catchments. Journal of Climate.

[24] Paulsen J, Körner C (2014). A climate-based model to predict potential treeline position around the globe. Alpine Botany.

[25] Lieth H (1972). Modelling the primary productivity of the world. Nature and Resources, UNESCO.

[26] Karger, D. N., Brun, P. & Zimmermann, N. E. CHELSA Climatologies at High resolution for the Earth Land Surface Areas. CHELSA V2.1: Technical specification. Tech. Rep., Swiss Federal Research Institute WSL (2021).

[27] Köppen, W. Das Geographische System der Klimate. In *Handbuch der Klimatologie in fünf Bänden*, 44 (Verlag von Gebrüder Borntraeger, Berlin, Germany, 1936).

[28] Peel MC, Finlayson BL, McMahon TA (2007). Updated world map of the Köppen-Geiger climate classification. Hydrology and Earth System Sciences.

[29] Wissmann, H. Die Klima und Vegetationsgebiete Eurasiens. Begleitworte zu einer Karte der Klimagebiete Eurasiens. *Zeitschrift der Gesellschaft für Erdkunde zu Berlin* 81–92 (1939).

[30] Thornthwaite CW (1931). The Climates of North America: According to a New Classification. Geographical Review.

[31] Troll, C. & Paffen, K. H. Karte der Jahreszeiten-Klimate der Erde (The Map of the Seasonal Climates of the Earth). *Erdkunde* **18**, 5–28. https://www.jstor.org/stable/25640079, accessed 2022-12-06 (1964).

[32] Fick SE, Hijmans RJ (2017). WorldClim 2: New 1-km spatial resolution climate surfaces for global land areas. International Journal of Climatology.

[33] Karger DN, Lange S, Hari C, Reyer CPO, Zimmermann NE (2021). CHELSA-W5E5 v1.1: W5E5 v1.0 downscaled with CHELSA v2.0.

[34] Funk C (2015). A global satellite-assisted precipitation climatology. Earth System Science Data.

[35] Zomer, R. & Trabucco, A. Global Aridity Index and Potential Evapotranspiration (ET0) Database: Version 6. https://figshare.com/articles/dataset/Global_Aridity_Index_and_Potential_Evapotranspiration_ET0_Climate_Database_v2/7504448/6 (2022). 10.1038/s41597-022-01493-1 PMC9287331 35840601

[36] Zomer RJ, Xu J, Trabucco A (2022). Version 3 of the Global Aridity Index and Potential Evapotranspiration Database. Scientific Data.

[37] Berghuijs WR, Woods RA, Hrachowitz M (2014). A precipitation shift from snow towards rain leads to a decrease in streamflow. Nature Climate Change.

[38] Hall, D. K., Salomonson, V. V. & Riggs, G. A. Modis/terra snow cover daily l3 global 500 m grid. Tech. Rep., NASA National Snow and Ice Data Center Distributed Active Archive Center, Boulder, Colorado, USA. 10.5067/MODIS/MOD10A1.006. Version 6 (2016).

[39] Salomonson VV, Appel I (2004). Estimating fractional snow cover from modis using the normalized difference snow index. Remote Sensing of Environment.

[40] Tang, Z. *et al*. Satellite observed spatiotemporal variability of snow cover and snow phenology over high mountain asia from 2002 to 2021. *Journal of Hydrology* **613**, 10.1016/j.jhydrol.2022.128438 (2022).

[41] Buchhorn M (2020). Zenodo.

[42] Buchhorn M (2020). Zenodo.

[43] RGI Consortium. Randolph Glacier Inventory – A Dataset of Global Glacier Outlines: Version 6.0, 10.7265/4m1f-gd79 (2017).

[44] Erasov NV (1986). Method for determining of volume of mountain glaciers. Mater. Glyatsiol. Issled.

[45] Hugonnet R (2021). Accelerated global glacier mass loss in the early twenty-first century. Nature.

[46] Olson, S. A. & Williams-Sether, T. Streamflow Characteristics at Streamgages in Northern Afghanistan and Selected Locations. Data Series 529, U.S. Department of the Interior, U.S. Geological Survey (2010).

[47] Shults, V. *Rivers of Middle Asia*, second edn (Gidrometeoizdat, Leningrad, 1965).

[48] Durre I, Menne MJ, Gleason BE, Houston TG, Vose RS (2010). Comprehensive Automated Quality Assurance of Daily Surface Observations. Journal of Applied Meteorology and Climatology.

[49] Harris I, Osborn TJ, Jones P, Lister D (2020). Version 4 of the CRU TS monthly high-resolution gridded multivariate climate dataset. Scientific Data.

[50] Huffman GJ, Stocker EF, Bolvin DT, Nelkin EJ, Jackson T (2019). GPM IMERG Final Precipitation L3 1 day 0.1 degree x 0.1 degree V06.

[51] Funk C (2015). The climate hazards infrared precipitation with stations— a new environmental record for monitoring extremes. Scientific Data.

[52] Yatagai A (2012). APHRODITE: Constructing a Long-Term Daily Gridded Precipitation Dataset for Asia Based on a Dense Network of Rain Gauges. Bulletin of the American Meteorological Society.

[53] Dilinuer, T. *et al*. Systematical Evaluation of Three Gridded Daily Precipitation Products Against Rain Gauge Observations Over Central Asia. *Frontiers in Earth Science* **9**, 10.3389/feart.2021.699628 (2021).

[54] Salehie O (2021). Ranking of gridded precipitation datasets by merging compromise programming and global performance index: A case study of the Amu Darya basin. Theoretical and Applied Climatology.

[55] Wang S (2022). Assessing Gridded Precipitation and Air Temperature Products in the Ayakkum Lake, Central Asia. Sustainability.

[56] Zandler H, Haag I, Samimi C (2019). Evaluation needs and temporal performance differences of gridded precipitation products in peripheral mountain regions. Scientific Reports.

[57] Peña-Guerrero MD, Umirbekov A, Tarasova L, Müller D (2022). Comparing the performance of high-resolution global precipitation products across topographic and climatic gradients of Central Asia. International Journal of Climatology.

[58] Zhang L, Potter N, Hickel K, Zhang Y, Shao Q (2008). Water balance modeling over variable time scales based on the Budyko framework – Model development and testing. Journal of Hydrology.

[59] Senay GB (2013). Operational Evapotranspiration Mapping Using Remote Sensing and Weather Datasets: A New Parameterization for the SSEB Approach. JAWRA Journal of the American Water Resources Association.

[60] Zhang Y (2019). Coupled estimation of 500 m and 8-day resolution global evapotranspiration and gross primary production in 2002– 2017. Remote Sensing of Environment.

[61] Elnashar A, Wang L, Wu B, Zhu W, Zeng H (2021). Synthesis of global actual evapotranspiration from 1982 to 2019. Earth System Science Data.

[62] Zambrano-Bigiarini M (2020). Zenodo.

[63] Sen PK (1968). Estimates of the regression coefficient based on kendall’s tau. Journal of the American Statistical Association.

[64] Becker A (2013). A description of the global land-surface precipitation data products of the Global Precipitation Climatology Centre with sample applications including centennial (trend) analysis from 1901– present. Earth System Science Data.

[65] Ragettli S (2020). Zenodo.

[66] Linke S (2019). Global hydro-environmental sub-basin and river reach characteristics at high spatial resolution. Scientific Data.

[67] Messager ML, Lehner B, Grill G, Nedeva I, Schmitt O (2016). Estimating the volume and age of water stored in global lakes using a geo-statistical approach. Nature Communications.

[68] Lehner B, Grill G (2013). Global river hydrography and network routing: Baseline data and new approaches to study the world’s large river systems. Hydrological Processes.

[69] Hersbach H (2020). The ERA5 global reanalysis. Quarterly Journal of the Royal Meteorological Society.

